# The Cold Shock Domain of YB-1 Segregates RNA from DNA by Non-Bonded Interactions

**DOI:** 10.1371/journal.pone.0130318

**Published:** 2015-07-06

**Authors:** Vladislav Kljashtorny, Stanislav Nikonov, Lev Ovchinnikov, Dmitry Lyabin, Nicolas Vodovar, Patrick Curmi, Philippe Manivet

**Affiliations:** 1 Institute of Protein Research, Russian Academy of Sciences, Pushchino, Moscow Region, 142290, Russia; 2 Institut National de la Santé et de la Recherche Médicale (INSERM), UMR 829, Laboratoire Structure-Activité des Biomolécules Normales et Pathologiques, Bd François Mitterrand, 91025 Evry Cedex, France; 3 Institut National de la Santé et de la Recherche Médicale (INSERM), UMRS 942, Hôpital Lariboisière, 41 boulevard de la Chapelle, 75475 Paris cedex 10, France; 4 Assistance Publique—Hôpitaux de paris (APHP), Hôpital Lariboisière, Service de Biochimie et de Biologie Moléculaire, Paris, France; 5 UBCS (Unité de Biologie Clinique Structurale)-Centre de Ressources Biologiques BB-0033-00064, 2 rue Ambroise Paré, 75475 Paris cedex 10, France; Institut National de la Santé et de la Recherche Médicale, FRANCE

## Abstract

The human YB-1 protein plays multiple cellular roles, of which many are dictated by its binding to RNA and DNA through its Cold Shock Domain (CSD). Using molecular dynamics simulation approaches validated by experimental assays, the YB1 CSD was found to interact with nucleic acids in a sequence-dependent manner and with a higher affinity for RNA than DNA. The binding properties of the YB1 CSD were close to those observed for the related bacterial Cold Shock Proteins (CSP), albeit some differences in sequence specificity. The results provide insights in the molecular mechanisms whereby YB-1 interacts with nucleic acids.

## Introduction

The eukaryotic Y-box binding protein 1 (YB-1) is a multifunctional DNA/RNA binding protein that participates in many cellular events through its binding to nucleic acids (NA) and other proteins (reviewed in [1,2,3]).

YB-1 is involved in different aspect of DNA biology. At a transcriptional level, YB-1 binds to the so-called Y-box that is present in the promoter regions of a large number of genes that are involved in cell division, differentiation, apoptosis, and immune and stress responses, and regulates their transcription in a positive or negative fashion (reviewed in [1,3]). YB-1 also displays a high affinity for single-stranded (ss) DNA, DNA damaged regions [4,5], and possesses a RNA and DNA melting and annealing activity [6,7], hence its involvement in DNA repair [8,9].

YB-1 also binds to RNA both in the nucleus and the cytoplasm. YB-1 binds to nascent mRNA [10,11] and is involved in the selection of alternative splice sites [12,13]. In the cytoplasm, YB-1 is associated with both translated and untranslated mRNAs and interferes with mRNA function and stability in a YB-1/mRNA ratio-dependent manner [14–18]: at low YB-1/mRNA ratio, YB-1 globally activates translation initiation while the saturation of mRNA by YB-1 blocks translation initiation and is accompanied by a complete cessation of protein synthesis [16,17]. An interplay between YB-1 and a series of other protein partners is however necessary to switch mRNA from an untranslated to a translated state. The displacement of YB-1 from mRNA and the stimulation of translation are facilitated by the poly(A) binding protein (PABP) which displays an enhanced affinity for mRNA when interacting with the translation initiation factor eIF4G [19]: the inhibition of translation initiation is caused by the competition between YB-1 and eIF4F for binding to the cap-adjacent region of mRNA [17,18]. Finally, it has been proposed that YB-1 is directly associated with the mRNA cap-structure and that this interaction may be regulated by YB-1 phosphorylation at Ser102 by Akt/PKB kinase [20,21].

**Fig 1:**
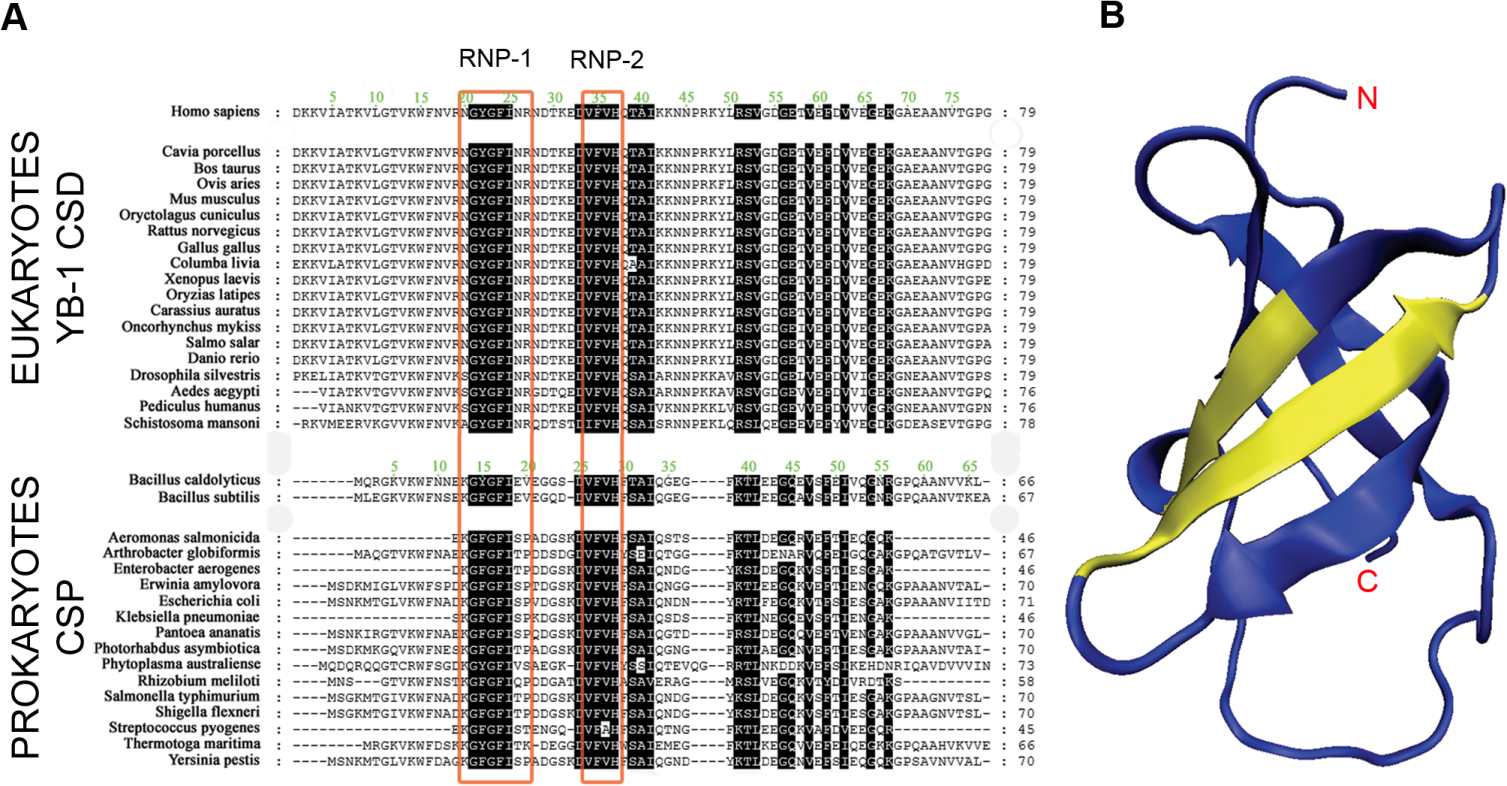
Sequence alignment of the CSDs from eukaryotic Y-box proteins and prokaryotic CSPs. **A.** The RNA-binding motifs are boxed in red; highly conserved amino acid residues (identity > 95%) are highlighted in black. **B.** 3D representation of the human CSD^YB-1^ structure. The RNP-1 and RNP-2 motifs are shown in yellow; N and C indicate the N- and C-termini of the domain.

YB-1 contains three domains: a N-terminal alanine/proline rich domain (A/P), an intermediate Cold Shock Domain (CSD) which comprises two RNA-binding motifs RNP-1 and RNP-2 that are conserved among eukaryotic YB-1 proteins and prokaryotic CSD proteins (CSP, **Fig 1A**), and a C-terminal domain which contains alternating positively and negatively charged amino-acid clusters. YB-1 is a rather difficult target for both NMR and X-ray structural methods due to its intrinsic instability that has hampered the solving of its overall structure so far. The only structural information available to date is the NMR structure of the CSD from human YB-1 (named hereafter CSD^YB-1^) [22]. The CSD^YB-1^ exhibits a highly conserved beta-barrel structure (**Fig 1B**), which has been described in many nucleic acids binding proteins [23]. Interestingly, bacterial CSPs consist of a single cold shock domain and are involved to low temperature adaptation [24].

The structural basis for the interaction of YB-1 with nucleic acids remains unclear. A biochemical study showed that the full-length YB-1 exhibit a preferential binding to G-rich DNA [25] and RNA [14] sequences while another supports that, similarly to the bacterial CSPs, the CSD^YB-1^ specifically binds to pyrimidine-rich sequences (T or C) [22]. It is worth noting that the affinity of the CSD^YB-1^ for oligonucleotides is much lower than that of CSPs [22]. This low affinity hinders biochemical assays and explains the absence of detailed information about the structure of CSD^YB-1^:oligonucleotide complexes. Such structure information is critical to understand the molecular mechanisms of the multiple nucleic acid-dependent functions of YB-1.

We thus propose here, for the first time, to use molecular dynamics simulation (MDS) methods to investigate the interactions of the CSD^YB-1^ with short single-stranded desoxyribo- and ribo-oligonucleotides. The results show that the CSD^YB-1^ preferentially binds to G-rich sequences as previously stated [14,25], with a higher affinity for RNA than for DNA. Furthermore, the MDS data unraveled the mechanistic basis for the preference of the CSD^YB-1^ binding to specific nucleic acid sequences. Finally, the good agreement between the present MDS results and experimental data available to date support MDS as a valuable approach to study the binding properties of nucleic-acid binding proteins.

## Methods

### Generation of the CSD^YB-1^:NA complex.

**Fig 2:**
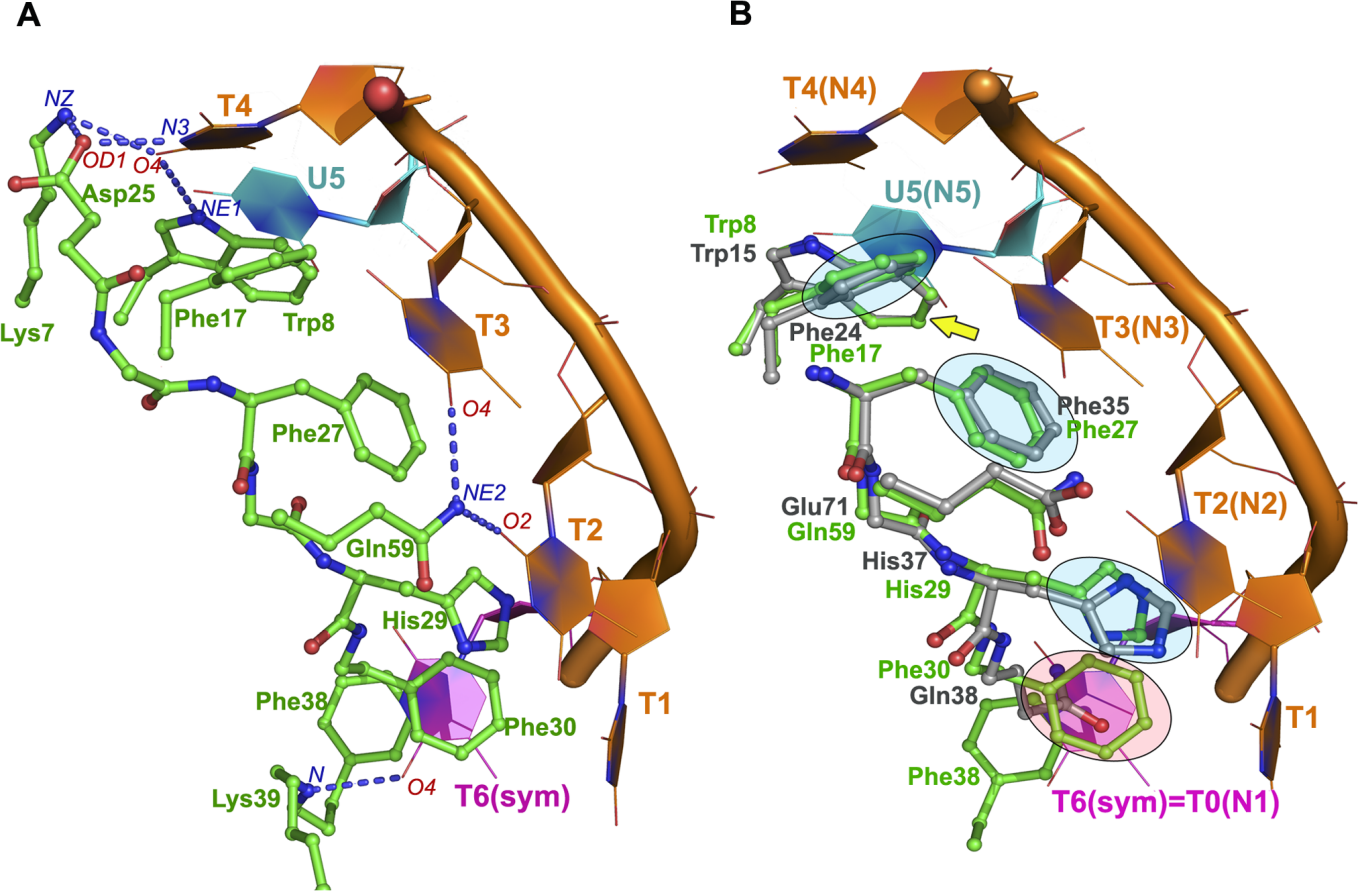
Superposition between the bacterial CSP and the CSD^YB-1^ nucleic acid-binding domains. **A**. Key interactions between the bacterial CSP (green) and an oligo(dT) (orange) obtained from the CSP:oligo(dT) crystal structure.[22] The intermolecular H-bond formed between the protein and the ssDNA are shown as blue dotted lines. **B.** Superposition of the CSD^YB-1^ (gray) with the CSP:dT6 complex (green and orange, respectively). The side chains of amino acids involved in stacking with nucleotide bases are highlighted in blue. These three binding sites, that are structurally equivalent between the bacterial CSP and the CSD^YB-1^ are labelled N1, N2 and N3. The nucleotide shown in magenta represents the symmetrically related molecule of the complex and shows the additional nucleotide binding site formed by Phe30 and Phe38 (binding site 1, N1), which is only present in the bacterial CSP.

The NMR structure of the human CSD^YB-1^ (PDB-entry 1H95) relaxed after 50 ns of MDS (see below the MDS protocol) was used as a starting model. This structure does not contain any nucleic acid as the interaction between the CSD^YB-1^:NA complex is not stable enough to be solved by NMR [22]. The NMR structure of CSD^YB-1^ [22] exhibits a β-barrel fold that is highly similar to the structures of the *Bacillus caldolyticus* Bc-CspB [26] and the *Bacillus Subtilis* Bs-CspB [27] CPSs, solved in the presence of single-stranded dT6 oligonucleotides (PDB-entries 2HAX and 2ES2, respectively). Indeed, from a structural standpoint, the nucleic binding sites of the CSD^YB-1^ and the aforementioned CSPs are highly conserved as illustrated by an RMSD value of 0.49Å for the 12 C_α_-atoms of the binding site (amino acids 15–18, 26–30, and 59–61 based on Bs-CspB numbering; **Fig 1A and 1B**). Given the structural similarities observed between the CSD^YB-1^ and the bacterial CSP, the coordinates of the oligonucleotide from the Bs-CspB:dT6 were used to build the CSD^YB-1^:DNA complexes used in this study. Of note, the 6^th^ residue of the dT6 oligonucleotide from the Bs-CspB:dT6 crystal was bound to symmetrically related Bs-CspB molecule (**Fig 2**, [26]). Therefore an additional T residue was added at 5'-terminus of the dT6 oligonucleotide to obtain the Bs-CspB:dT7 structure. Using the sugar-phosphate backbone of the oligo(dT) from the Bs-CspB:dT7, each nucleotide was substituted by A, C or G to obtain oligo(dA), oligo(dG) and oligo(dC). One additional nucleotide was added to each extremity, ending up with the CSD^YB-1^ bound to nonameric oligonucleotides. The polarity of oligonucleotide obtained in such a way and corresponding to the orientation of dT6 in the bacterial Bs-CspB:dT7 structure is referred as standard or *direct* orientation. In contrast to non-standard or *reversed* orientation where the strand is oriented upside down. To generate such conformation the atoms of the nucleotide bases were aligned to keep the stacking with the amino acid residues of CSD^YB-1^ whereas the atoms of sugar and phosphate groups were ignored. Thus, the reversed orientated oligonucleotide preserved the main contacts between nucleotide bases and amino acid residues and only had the flipped polarity of sugar-phosphate backbone.

Similarly, the CSD^YB-1^ complexes were generated for ssRNA oligonucleotides. The bacterial numbering scheme of nucleotides bound to the Bs-CspB was used in this work so that the side chains of His29 (His37 in CSD^YB-1^), Phe27 (Phe35 in CSD^YB-1^) and Phe17 (Phe24 in CSD^YB-1^) are referred as binding sites 2, 3 and 4, and the corresponding interacting bases of oligonucleotides are referred as N2, N3, N4 (**Fig 2B**) where N refers to each A, T/U, C and G. When referring to reversed orientation of oligonucleotide we add a little subscript “r” after indication the nucleotide (for instance, dG2r would indicate desoxyriboguanidine bound at the site 2 and having reversed polarity of sugar-phosphate backbone).

More recently, an additional 5^th^ binding site has been identified in two crystals of Bs-CspB in complex with short single-stranded RNA molecules (PDB entries 3PF4 and 3PF5 [28]). This 5^th^ site that involves the Trp8 residue (Trp15 in CSD^YB-1^) was not observed in the Bs-CspB:DNA complexes, as Trp8 was involved in crystal contacts [26]. The influence of this extra site was evaluated by MD simulations using the coordinate of the polyU oligonucleotide from the Bs-CspB:U6 complex as starting model for MDS. As both starting models gave similar results, only differences between the two setups will be further discussed when applicable.

The comparison of the CSD^YB-1^:dT9 and the Bs-CspB:dT7/U6 structures revealed the absence of some critical amino acid residues in CSD^YB-1^ that are involved in the binding of CSPs to nucleic acids. The lack of these amino acids in CSD^YB-1^ may contribute to the weak interaction of the CSD^YB-1^:NA complex as compared to that of CSPs:NA and may also explain the difficulty encountered when studying CSD^YB-1^:NA complexes by biochemical and computational methods such as MDS. MDS experiments consist first in equilibrating the system using position restrains before running simulations under relaxed conditions. In the present study, due to the CSD^YB-1^:NA complexes instability, data acquired starting from the first nanoseconds of the simulation were considered in order to determine the molecular basis for such an instability in CSD^YB-1^:NA complexes.

### Molecular dynamics simulations

MDS were performed with the Gromacs 4.5 software [29] using the Charmm27 force field [30,31]. The starting models of the complexes were placed in an orthogonal water box of 80Å x 60Å x 60Å that contained about 10 000 water molecules of TIP3 type [32]. The water box was generated in the way to keep at least 15Å of bulk water between the surface of the complex and the edge of the water box.

The protonated state for residue was set up as follows: i) the acid functions of acidic residues were deprotonated and carried a -1 charge; ii) the amine functions of basic residues were deprotonated and carried a +1 charge; iii) the phosphate groups of nucleotides were deprotonated and carried a -1 charge. The system was neutralized by the addition of 6 sodium ions to neutralize the -6 charge of the complexes (+3 for the CSD^YB-1^ and -9 for an oligonucleotide). Prior to MDS production, the systems were i) minimized to the nearest local minimum using the steepest descent algorithm, ii) equilibrated at a temperature of 310 K for 1 ns with a positional restrains applied to the solute atoms, unless otherwise stated. The systems were then subjected to 50 ns MDS, at constant pressure and temperature (NPT), using a 1 fs integration time-step. The following parameters for NPT MDS were used: i) bond-length were only constrained for water molecules, ii) the long-term non-bonded interactions were evaluated using the PME algorithm, iii) the temperature and pressure were controlled using the velocity rescaling method [33] and the Berendsen algorithm [34], respectively. Coordinates were saved every 0.1 ps. For each the complexes, trajectories were performed 3 times independently.

As mentioned above, prior to simulate the different CSD^YB-1^:NA complexes, the CSD^YB-1^ alone was relaxed after 50 ns of MDS by using the same system and MDS protocol as mentioned just here above. Three chlorine ions were used for neutralizing the system.

In order to evaluate the repeatability of our results we performed MDS in triplicate for each CSD^YB-1^:NA complexes by assigning random velocities. No significant differences were obtained for all complexes among the replicates.

### MSD trajectory validation

To validate the trajectories, the NMR data available at BioMagResBank (entry number 4147 [35]) were used. The comparison between experimental and calculated chemical shifts was achieved with Sparta software [36] using C_α_- and C_β_-atoms (**S1 Fig**). The stereochemical parameters of the models at the different steps of the trajectories were also examined with the Procheck packages [37]. An example of Ramachandran plot is provided in **S2 Fig**.

### Geometric parameters of the CSD:oligonucleotide interactions

The interactions between the CSD^YB-1^ and NA can be described in polar and non-polar terms. The former include H-bonds and electrostatic interactions while the latter involve Van-der-Waals contacts and stacking of aromatic groups. The protein:NA and NA:NA H-bonds as well as the stacking interactions between nucleotides and amino acids were evaluated for each kind of nucleotide at positions 2 to 4 (**Fig 2B**).

H-bonds were scored at each snapshot of the trajectories when the distance between appropriate H-bond donors and acceptors was less than 3.5Å and the angle acceptor–hydrogen donor was within 30 degrees. Total number of H-bonds between CSD and each oligonucleotide was counted for each snapshot every 10 ps. For each H-bond, we additionally calculated its occupancy (Occ), *i*.*e*. the percentage of snapshots along the trajectory where the H-bond was scored. To that end, next expression was applied Occ = N/T, where N is the number of snapshots where the H-bond was scored, and T is the total number of snapshots.

Another important parameter to take into consideration for H-bonds is their accessibility to the solvent molecules (SA, Å^2^): the less accessible to the solvent an H-bond is, the more it contributes to the stability of the complex [38]. The solvent accessibility for each atom was determined using the *g_sas* utility from the Gromacs package [29]. Thus, the relative strength of an H-bond depends on both its occupancy and its accessibility to water molecules.

To evaluate stacking interactions, the two following criteria were used: i) the average distance between the geometric centers of the rings of both the base and the aromatic residue during the trajectories, and ii) the angle between the normal of the planes of the rings of the stacked base and the aromatic residue. Well stacked rings show an average distance around 4.2–4.5Å, and an angle between 0 and 30 degrees throughout the trajectory. These two parameters were also evaluated for each snapshot every 10 ps along the trajectory for each of the complex.

For both H-bond numbers and stacking parameters, a total of 15000 measurements (5000 snapshots from each of 3 trajectories) were collected and pooled for each complex.

### Energetic parameters of the CSD:oligonucleotide interactions

To evaluate binding free energies and to make a ranking for the CSD:NA complexes, the Molecular Mechanics/Poisson-Boltzmann Surface Area (MM/PBSA) method [39] was used. According this method the binding free energy (ΔG_bind_) is calculated as follows:

$$\Delta G_{Bind}=\Delta H-T\Delta S\approx \Delta E_{MM}+\Delta G_{sol}-T\Delta S$$ (1)

$$\Delta E_{MM}=\Delta E_{electrostatic}+\Delta E_{vdw}$$ (2)

$$\Delta G_{sol}=\Delta G_{PB}+\Delta G_{SA}$$ (3)

where ΔE_MM_, ΔG_sol_ and-TΔS are the changes of the gas phase Molecular Mechanics energy, the solvation free energy, and the conformational entropy upon binding, respectively. ΔE_MM_ includes ΔE_electrostatic_ (electrostatic), and ΔE_vdw_ (van der Waals) energies. ΔG_sol_ is the sum of ΔG_PB_, the electrostatic solvation energy (polar contribution), and ΔG_SA_, the non-electrostatic solvation component (non-polar contribution). The polar contribution was calculated here by solving the finite-difference Poisson-Boltzmann equation model using the *PBEQ* module of CHARMM [40]. The grid spacing was set to 0.4 Å, and the longest linear dimension of the grid was extended at least 50% beyond the system; the value of the exterior dielectric constant was set to 80, and the solute dielectric constant was set to each of 1, 2 and 4. The non-polar energy ΔG_SA_ was estimated using solvent accessible surface (SAS) as 0.00542 × SAS + 0.92 kcal.mol^-1^ [41]. For the calculation of ΔG_PB_ and ΔG_SA_, 50 snapshots (each 1 ns) evenly extracted from the entire MDS trajectories for each complex (0 to 50 ns) were used. ΔE_MM_ was calculated based on 5000 snapshots from 0 to 50 ns. The conformational entropy change-TΔS was calculated by quasi-harmonic approach using the *VIBRAN* module of CHARMM [40]. 500000 snapshots extracted from single trajectory were used to evaluate entropic contribution. The calculation for each complex was based on three trajectories and the final value of ΔG_bind_ was averaged over 3 measurements.

Calculation methods to measure the binding energy of a system were shown to be dependent on many parameters including the system itself, the simulation protocol, the length of the trajectory, the force-fields, and the solute dielectric constant, and often yield to wrong absolute values [39]. To limit these discrepancies, the relative binding energy of each protein:NA with respect to the most stable of the protein:NA complex (OligoG, ΔΔG_bind_ = ΔG_bind (oligoN)_—ΔG_bind (oligoG)_) was considered, instead of the absolute binding energy for each protein:NA complex. As for such complex and unstable systems, the sole consideration of relative binding energies may draw biased conclusion, the combination of visual inspection, the averaged parameters of key contacts and MM/PBSA calculations was also used to confidently evaluate the stability of the different protein:NA complexes.

### Biochemical procedures

Recombinant YB-1 and recombinant CSD proteins were purified as described previously [42]. For the electrophoretic mobility shift assay (EMSA), 7 pmol of chemically synthesized RNA (5’-AGGAUGGGUGAGUGAGGUAG-3’) or DNA (5’-AGGATGGGTGAGTGAGGTAG-3’) oligonucleotides (“DNA-synthesis”, Russia) were incubated with increasing amounts of recombinant YB-1 (0.35, 0.7, 1.4, 2.8 μM) or CSD (3, 6, 12, 24 μM) in 10 μl reaction buffer (10 mM Hepes-KOH, pH 7.6, 100 mM KCl) at 30°C for 15 minutes. RNA- and DNA-protein complexes were analysed by 8% native polyacrylamide gel electrophoresis (PAGE) in TBE buffer followed by silver staining. Band intensities for free RNA or DNA were determined by gel scanning and quantification using ImageJ software [43].

### Statistical analysis

Data are presented by box plots and include a mean +/- standard deviation or a median and interquartile range, when appropriate. Statistics were performed using the R package (http://www.r-project.org/). Analyses of variance (ANOVA) or pairwise t-test were used to comparison the data for the different CSD^YB-1^:NA complexes. A two sided type I error of 0.05 was applied with using Bonferroni correction for multiple tests [44] when appropriate.

## Results and Discussion

### The CSD^YB-1^ and the bacterial CSPs exhibit different NA binding sites

The CSD^YB-1^ and bacterial CSPs belong to the family of the cold shock proteins that exhibit a beta barrel fold that includes two highly conserved RNA-binding motifs, RNP-1 and RNP-2 (**Fig 1A**), which include most of the residues involved in the interactions with nucleic acids. Both the beta barrel structure and the RNP-1/RNP-2 motifs are highly conserved among CSPs. This structural similarity extends to the CSD domain of YB-1 as illustrated by a 0.49Å RMSD when the structures of the Bc-CspB and the CSD^YB-1^ were superposed. Contrary to the bacterial CSPs, the structure of the CSD^YB-1^ was not determined in the presence of any nucleic acids. Given the close structural relationship between the CSD^YB-1^ and Bs-CspB, their superposition was used to assign the nucleotide binding sites in the CSD^YB-1^. This analysis showed that the nucleotide binding site of the CSD^YB-1^ resembles to that of the CSPs, albeit some notable differences: i) the Phe30 residue of the Bs-CspB that is involved in nucleotide stacking is substituted by the non-aromatic Gln38 in CSD^YB-1^, ii) the Gln59 of Bs-CspB that is involved in H-bond with an oligonucleotide base is substituted by Glu71 in CSD^YB-1^; and, iii) most of the bacterial CSPs contain an Phe residue at location 38 which is also stacked to a nucleotide bases, while it is replaced by a Gln in the CSD^YB-1^ (**Figs 1 and 2**; of note, in the crystal structure of the Bs-CspB, this residue interacts with symmetrically related molecule shown in magenta). Consequently, the bacterial CSPs and the CSD^YB-1^ share 3 to 4 structural nucleotide binding sites (**Fig 2**, N2-N4, N5 was detected only for Bs-CspB:U6 in the crystal structure) while bacterial CSPs exhibit an additional binding site (**Fig 2**, N1), which is absent in the CSD^YB-1^. Therefore, the nucleotide at position 1 that is located between binding sites N1 and N2 in the bacterial CSPs does not make specific interactions with the binding site N2 of the CSD^YB-1^ except by non-specific Van-der-Waals contacts. Altogether, these observations suggest that the differences between the binding sites of bacterial CSPs and the CSD^YB-1^ may affect both the binding specificity and the affinity for nucleic acids of CSD^YB-1^ as compared to CSPs.

### The CSD^YB-1^ and the CSPs exhibit different binding sequence specificity

To evaluate the binding properties of the CSD^YB-1^ to nucleic acids, the interaction between the CSD^YB-1^ and different homo-oligonucleotides was studied by MDS. Analyses of the MDS trajectories were focused on the three binding sites (N2-N4) that are structurally equivalent in the bacterial CSPs and the CSD^YB-1^. These three sites have been shown to determine the binding specificity of bacterial CSPs to nucleic acids [26,27] and are also expected to govern the binding specificity of CSD^YB-1^ to nucleic acids. MDS experiments involving the CSD^YB-1^ associated with oligo(dA), oligo(dT), oligo(dC) or oligo(dG) showed that the CSD^YB-1^ binds preferentially to the oligo(dG) sequence providing the lowest ∆G values and the most stable intermolecular contacts (**Table 1**). Significant stacking interactions were also observed for oligo(dA) especially in binding sites N2 and N3: the median distance between adenine bases and CSD^YB-1^ aromatic residues were 5.1Å and 4.8Å, respectively; the median angle between ring planes and CSD^YB-1^ aromatic residues were 30° and 23°, respectively (**Table 1**).

**Table 1:** Different types of interactions between CSD^YB-1^ and oligonucleotides. For the parameters of stacking median value during the three trajectories as well as the first and the third quartiles of the data distribution are given. For the H-bond parameters the mean plus or minus a standard deviation are shown.

Nucleic acid	Nucleotide	Pos2		Pos3		Pos4		Number of NA-CSD H-bonds	Number of NA-NA H-bonds	ΔΔG_Bind_ kcal/mol
		Distance, Å	Angle, deg	Distance, Å	Angle, deg	Distance, Å	Angle, deg			
DNA	dG9	4.9 (4.6–5.3)	27 (18–41)	4.2 (3.7–7.8)	17 (9–56)	7.4 (6.9–7.9)	79 (70–85)	7.1 ± 2.1	2.0 ± 1.7	0
	dA9	5.1 (3.9–6.6)	30 (15–53)	4.8 (3.9–7.3)	23 (9–66)	8.4 (5.5–9.6)	71 (53–82)	5.2 ± 2.2	1.6 ± 1.2	67
	dT9	4.8 (4.0–11.4)	26 (15–46)	6.8 (4.2–7.7)	23 (13–37)	13.1 (6.4–16.5)	64 (45–78)	5.0 ± 2.1	1.1 ± 0.8	54
	dC9	7.6 (3.8–12.8)	29 (16–49)	8.4 (6.8–13.5)	54 (30–71)	12.3 (8.9–16.4)	69 (51–81)	5.4 ± 2.2	2.1 ± 1.3	23
	dGGTr	7.1 (5.3–12.0)	48 (33–63)	5.2 (4.0–7.9)	26 (14–50)	16.0 (14.0–17.6)	49 (29–69)	6.4 ± 3.4	3.5 ± 2.3	35
RNA	G9	4.8 (4.5–5.1)	20 (14–27)	4.3 (3.7–7.9)	23 (10–66)	6.6 (5.7–7.7)	76 (63–83)	6.7 ± 2.7	7.1 ± 2.2	0
	U9	8.6 (6.0–12.0)	42 (27–61)	5.4 (3.8–8.0)	22 (11–41)	5.0 (4.0–7.1)	40 (21–67)	3.9 ± 1.7	6.4 ± 2.0	-11
	A9	4.3 (4.0–4.7)	13 (8–19)	6.4 (5.1–8.6)	10 (5–38)	7.5 (6.9–7.9)	80 (71–85)	4.2 ± 1.9	5.1 ± 1.7	2
	C9	6.5 (3.8–7.3)	32 (18–45)	4.0 (3.8–4.2)	13 (8–19)	9.2 (6.0–12.0)	50 (35–68)	4.6 ± 1.9	7.0 ± 1.9	29
	GGUr	5.2 (4.7–24.6)	44 (34–54)	4.3 (3.9–20.2)	17 (10–38)	13.5 (10.7–16.2)	64 (46–77)	5.9 ± 2.7	9.9 ± 2.4	8
	U9r	12.4 (4.8–22.8)	50 (26–72)	18.6 (5.9–22.3)	52 (32–71)	19.9 (9.9–23.5)	53 (34–72)	5.6 ± 2.7	5.3 ± 1.5	30

**Fig 3:**
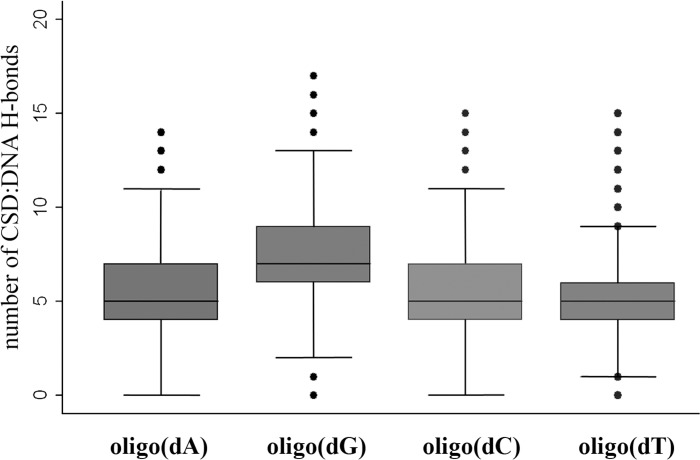
Number of intermolecular H-bonds in the complexes of CSD with different desoxyribooligonucleotides.

In contrast, the CSD^YB-1^:oligoT and the CSD^YB-1^:oligoC complexes were found significantly less stable with median distances between bases and CSD aromatic residues > 7 Å with only exception of dT at site N2 (distance = 4.8Å, angle = 26°). In particular, in the CSD^YB-1^:oligo(dC) complex, initial contacts at key positions were lost within the first nanoseconds of the MDS although non-specific interactions prevented the complex to dissociate. All the complexes with oligo(dA), oligo(dT), oligo(dC) had much higher ΔG_bind_ values compared with oligo(dG) which was driven not only by weaker stacking interactions but also by a total number of intermolecular hydrogen bonds. Thus, the average number of H-bonds between CSD and oligo(dG) was 7.1 whereas in the other complexes this number varied from 5.0 to 5.4 (**Fig 3**). The t-tests for the mean comparisons for the number of intermolecular H-bonds between different complexes revealed the statistically significant difference between all the groups (P-value < 0.001). However the difference was only biologically meaningful when compared CSD^YB-1^:oligo(dG) with other complexes. The Cohen’s D value [45] for these comparisons ranged between 0.8 and 1 standard deviation with absolute difference of about 2 H-bonds. Such a difference could correspond to a two-folds increase in affinity of CSD^YB-1^ for oligo(dG) compared to other oligonucleotides. Among other oligonucleotides oligo(dA) seems to be slightly more preferable ligand for CSD^YB-1^ followed by oligo(dT).

**Fig 4:**
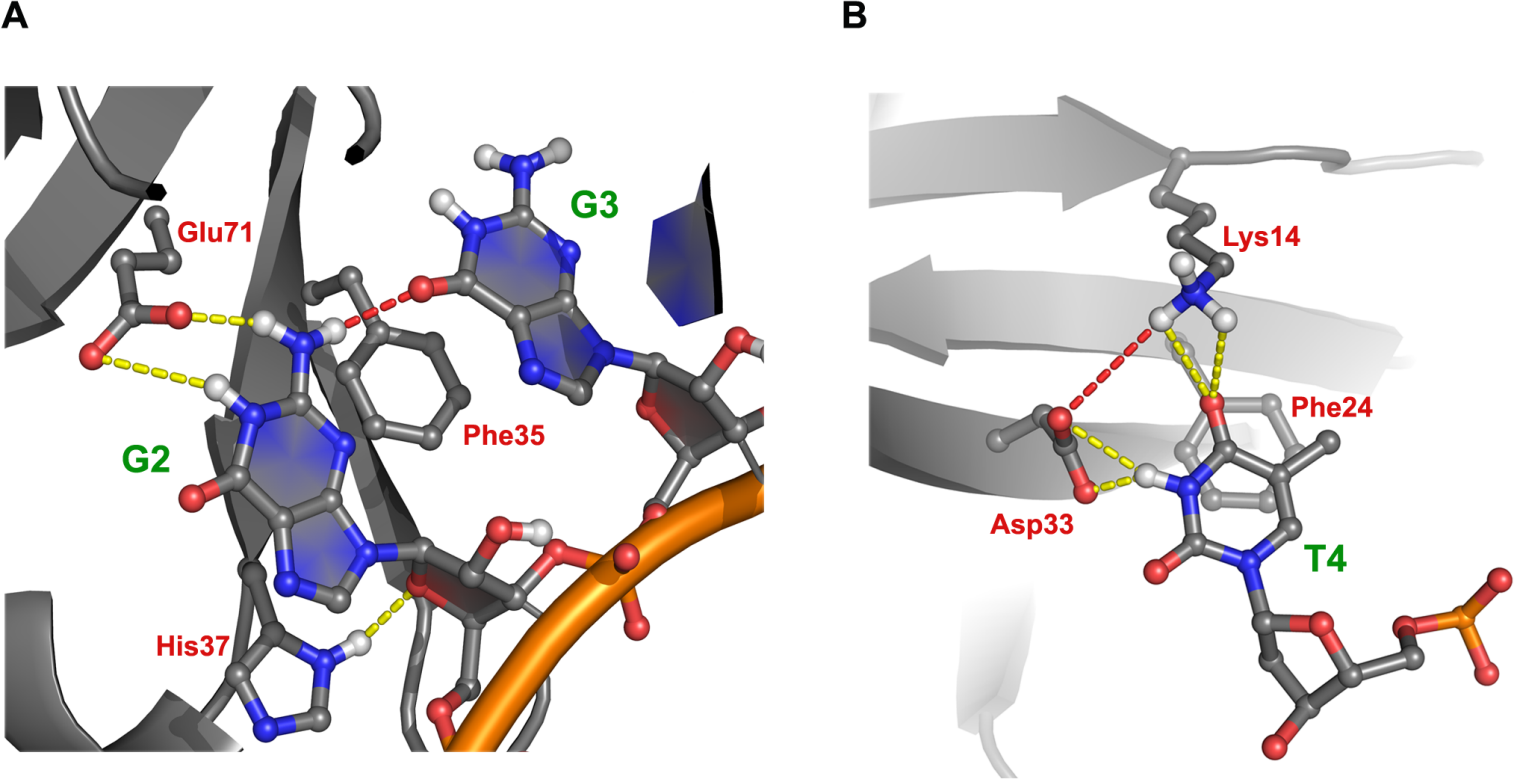
The CSD^YB-1^ preferentially binds to GGT sequence. **A.** Binding of the CSD^YB-1^ to oligo(dG) at positions 2 and 3 involves the formation intermolecular H-bonds (yellow dotted lines), including specific H-bonds between G2 and Glu71. In addition, specific intramolecular H-bonds (red dotted lines) between G2 and G3 were also observed. **B.** Binding of the CSD^YB-1^ to oligo(dT) at position 4 involves stacking interaction with Phe24 and the formation intermolecular H-bonds (yellow dotted lines) with Asp33 and Lys14. In addition, the interaction of oligo(dT) and the CSD^YB-1^ promotes the formation of an intramolecular H-bond between Asp33 and Lys14 (red dotted line).

Contrary to the CSD^YB-1^, bacterial CSPs exhibit a higher affinity for T-rich sequences [26,27]. The present results show that this specificity is mainly determined by the *NH* group of Lys39 main chain and the *NE2* atom of Gln59 side chain of Bs-CSP, which form strong H-bonds with the *O4* and *O2* atoms of thymidine at the N1 and N2 sites, respectively (**Fig 2A**). In the CSD^YB-1^, the N1 site is absent, and Gln59 is substituted by Glu71 at the N2 site. This Gln to Glu substitution at the N2 site completely changes the specificity of the CSD^YB-1^ where only a guanine can bind strongly and specifically at this position. This interaction is favored by the formation of strong H-bonds between the side chain of Glu71 and the nitrogen atoms *N1* and *N2* of dG2 (**Fig 4A**). These H-bonds had an average occupancy > 60%, which was also accompanied by a lower solvent accessibility of the respective atoms, strengthening these interactions. In addition, the formation of transient H-bonds between dG2 and dG3 was observed along the simulation (average occupancy of 25% over the 3 trajectory replicates) which may favor the binding of a guanine at the position N2.

Stable stacking between adenines at positions 2 and 3 and His37 and Phe35 was also detected during the trajectory (**Fig 4A**). The stacking of dA3 was accompanied with a persistent hydrogen bond between its *N6* atom and the main chain oxygen atom of Ala70 (average occupancy of 25% over the 3 trajectory replicates) (**S1 Table**). However, as mentioned above, the ∆G of the CSD^YB-1^:oligo(dA) complex was higher than the ∆G of the CSD^YB-1^:oligo(dG) complex, suggesting that the binding of guanines at the N2 and N3 sites is favored over the binding of adenines.

The N4 binding site is almost identical between bacterial CSPs and the CSD^YB-1^ (**Fig 2B**). In agreement with crystallographic data, the MDS results showed some specific H-bonds between the CSP Asp25—Lys7 pair and the polar atoms of the dT4-ring (**Fig 2A**). In the CSD^YB-1^ however, while similar contacts between the Asp33—Lys14 pair and dT4 polar atoms were observed, the interaction was marginally stable (occupancy about 5% over 3 trajectories) compared with the bacterial proteins (**Fig 4B**).

As in our MDS we observed disruption of most of the complexes, it is logic that CSD^YB-1^ involved in the most stable complex should possess lower flexibility compared to less stable ones. To test this hypothesis, we calculated the average atomic fluctuation (RMSF) for the CSD^YB-1^ residues and compared them among different complexes (**S3**and **S4 Figs**). The RMSFs for each amino acid residue were calculated based on 50000 snapshots (each 1 ps apart) and averaged over 3 trajectories. Cohen’s D effect size for all the comparisons were statistically significant (**Table 2**). The final rank of the flexibility of CSD^YB-1^ amino acid residues can be expressed as follows: Oligo(dG) < Oligo(dA) < Oligo(dT) << Oligo(dC). RSMD calculations on the same CSD^YB-1^:oligo complexes confirmed the same rank with a net marked higher flexibility for dC oligos (**S5 and S6 Figs**).

**Table 2:** Effect size by Cohen’s D for the pairwise comparison of the CSD residues RMSF in the complex with different oligonucleotides.

Oligonucleotide	dG9	dC9	dA9	dT9
dG9	-	-1.30*	-0.34*	-0.55*
dC9	1.30*	-	1.00*	0.73*
dA9	0.34*	-1.00*	-	-0.24*
dT9	0.55*	-0.73*	0.24*	-

* Indicate statistically significant difference on the significance level of 0.05.

Altogether, these MDS results showed that, in contrast to CSPs that bind preferentially T-rich sequences, the CSD^YB-1^ preferentially binds to G-rich sequences. This binding specificity of the CSD^YB-1^ is in agreement with the few experimental data available that showed that full-length YB-1 preferentially binds to G-rich sequence [25]. Detailed analysis predicts a GGT triplet to display the highest affinity. These data support a model whereby, the specificity of YB-1 binding to nucleic acid is mainly determined by its CSD domain, while the A/P rich and C-terminal domains may contribute to increase the stability of YB-1:NA complexes [43]. Finally, as previously observed experimentally [22], the present MDS analysis of the CSD^YB-1^:oligo(dG) interaction was found of lower affinity than that measured for the Bs-CSP:oligo(dT) complex. This reduced affinity is due to the loss of the N1 binding site in the CSD^YB-1^, as the N1 site present in the bacterial CSPs promotes strong and specific interactions with nucleic acids.

### The CSD^YB-1^ binds RNA with higher affinity than DNA

**Fig 5:**
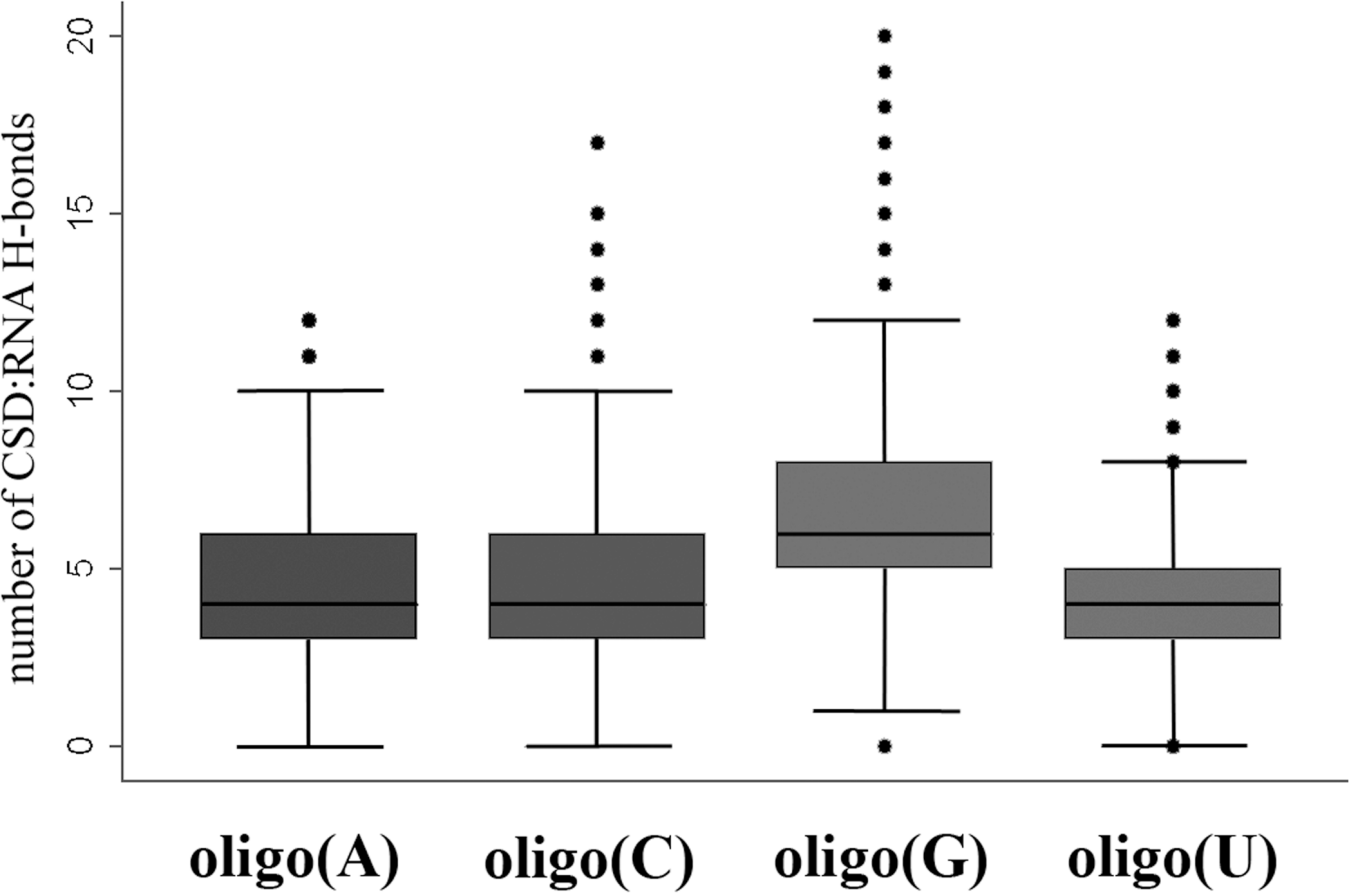
Number of intermolecular H-bonds in the complexes of CSD with different ribooligonucleotides.

The relative affinity of the CSD^YB-1^ for DNA and RNA was next examined. To this end MD simulations were extended to CSD^YB-1^:oligo(N) complexes. Comparing the RMSD plots for DNA and RNA show clearly that DNA complexes are fluctuating within a larger range of RMSD value pointing a higher flexibility for DNA than RNA (**S5 and S6 Figs**). As observed for the CSD^YB-1^:DNA interactions, a more stable binding of the CSD^YB-1^ was found for the oligo(G) with perfect stacking parameters especially at binding sites 2 and 3 (**Table 1**) and statistically significant (P-value < 0.001) higher average number of H-bonds between the protein and oligo(G) (**Fig 5**). Thus, the average number of H-bonds between CSD and oligoG was 6.7 which 0.8 to 1.2 standard deviations higher compared with other CSD^YB-1^:RNA complexes with absolute difference of above 2 H-bonds. Again such a difference could correspond to two folds of higher affinity of CSD for oligoG. The complex CSD^YB-1^:oligoG was used as a reference for ΔΔG_bind_ calculation.

Some of the interactions were such unstable that the complex dissociated within the first 5–10 ns of the MDS. Accordingly, we observed increasing fluctuations of the protein residues associated with a decreasing number of CSD:RNA contacts (**S3 Fig**): the highest fluctuations were observed for the least stable complex CSD^YB-1^:oligoC, although the Cohen’s D effect size was moderate (**Table 3**). Combining the data of different analyses we could rank the oligoribonucleotides by their affinity for CSD^YB-1^ as follows: OligoG > OligoA, OligoU > OligoC.

**Table 3:** Effect size by Cohen’s D for the pairwise comparison of the CSD residues RMSF in the complex with different oligonucleotides.

Oligonucleotide	G9	C9	A9	U9
G9	-	-0.23*	0.03	-0.27*
C9	0.23*	-	0.27*	-0.04
A9	-0.03	-0.27*	-	-0.30*
U9	0.27*	0.04	0.30*	-

* Indicate statistically significant difference on the significance level of 0.05.

Similarly to what observed with the CSD^YB-1^:oligo(dG) complex, we found specific intra-molecular H-bonds between the *N2* of G2 and the *O6* of G3 (**Fig 4A**). These H-bonds were found more stable in the case of RNA when compared to DNA in average along the three trajectories. Once again, this specific interaction between guanine at position 2 and 3 suggests a possible cooperative binding of these nucleotides to the N2 and N3 sites.

Regarding the N4 site, in contrast to DNA, the CSD^YB-1^ exhibited more favorable contact with U at position 4 with median distance between U4 and Phe24 of 5.0 Å (**Table 1).** This stacking is accompanied with the formation of a very strong H-bonds with the Asp33—Lys14 pair (**Fig 4B**); these H-bonds were significantly less stable when a thymidine (dT4) was stacked at the same position, *i*.*e*. these two H-bonds were only detected at the beginning of the MDS. Despite this difference at site N4, the binding of G2 and G3 to the amino acids of the N2 and N3 sites did not differ significantly between oligo(dG) and oligo(G).

**Fig 6:**
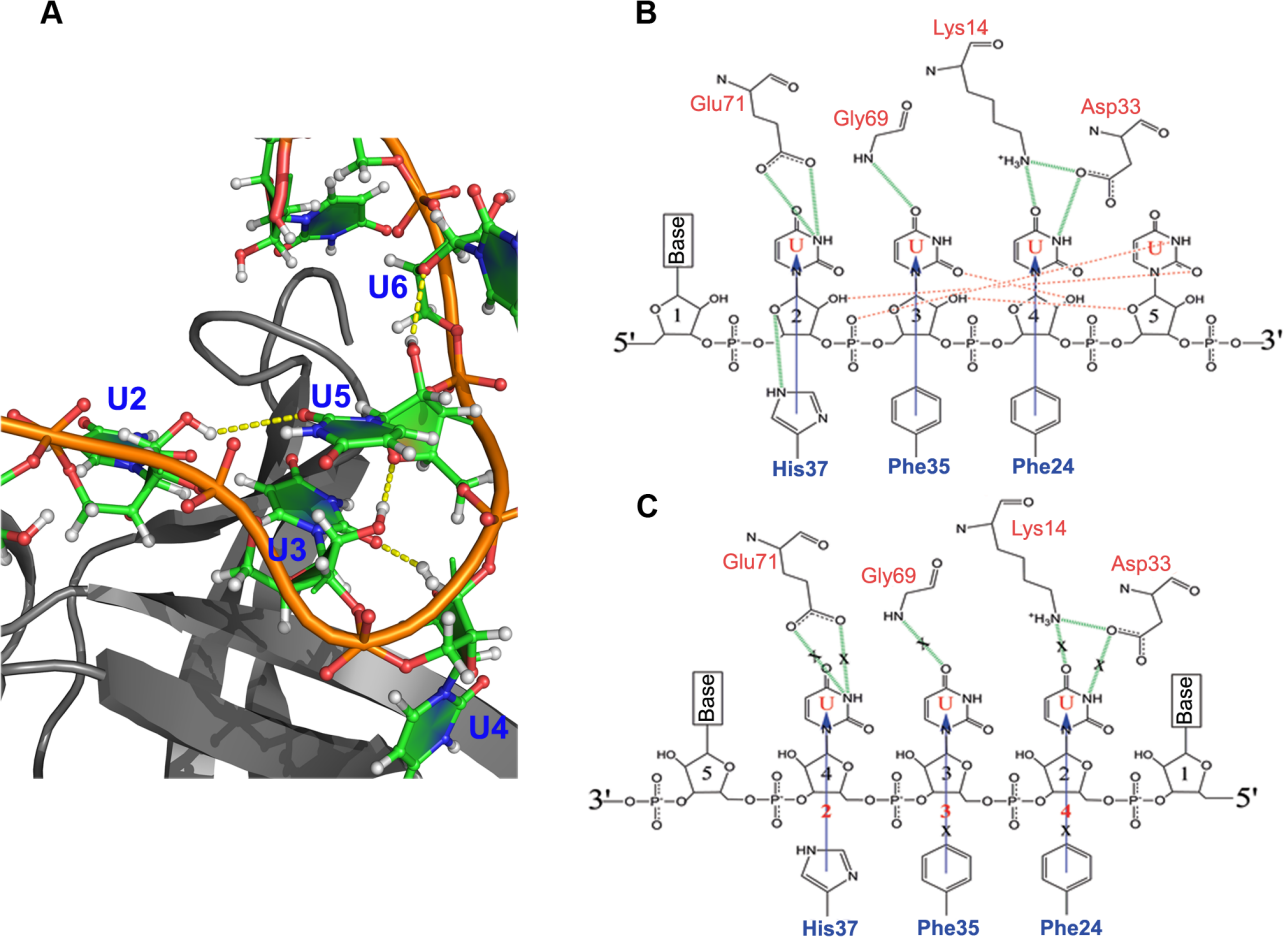
The CSD^YB-1^ binds preferentially to direct-oriented RNA. **A**. RNA-binding on the surface of CSD (grey) bound to an oligo(U) (green). Intramolecular H-bonds formed between ribonucleotides (yellow dotted lines) stabilise the local conformation of RNA when bound to the CSD. The CSD is shown in grey, the backbone of the oligonucleotide is shown in orange and the bases are shown in green. Nucleotides numbering is indicated. This H-bond network is not formed within DNA molecules. **B** and **C**. Planar representation of the interaction between CSD and direct- (**B**) or reverse-oriented (**C**) oligoribonucleotides. Regardless the orientation of the oligonucleotide, intermolecular H-bonding (green lines) between amino acids and ribonucleotides is identical, with the exception of the H-bond formed between His37 and U2 only observed for direct oligo(U) (**B**). The binding of the CSD^YB-1^ to a direct-oriented oligo(U) promotes the formation of intra-oligonucleotide H-bonding (red dashed line) that stabilises the interaction (**B**, H-bonds correspond to **A**). However, this intramolecular network is not formed when CSD binds to the reverse-oriented oligonucleotide (C) Consequently, this interaction is less favourable as illustrated by the weakening of the stacking (blue arrow) interaction between amino acids and nucleotides at positions 2 and 3 (marked with X).

The CSD^YB-1^:oligo(G) interaction was found more stable when compared to the CSD^YB-1^:oligo(dG) interaction (**S2 Table**). Deoxyribonucleotides differ from ribonucleotides by the lack of an alcohol group at the position 2' of the ribose. We found that this extra-OH groups present in RNA molecules were involved in both CSD^YB-1^:RNA and intra-RNA interactions that were obviously not observed for DNA. In particular, several stable H-bonds were observed between the CSD^YB-1^ residues Asn17, Tyr22, Gln38, Glu65, Gly66 and Lys68 and the ribose atoms at position 0, 1, 5, 6 and 8 (**S3 Table**). In addition, specific RNA intra-molecular H-bonds that involved the 2'-OH groups of the ribose of nucleotides 2 to 5 were detected (**Fig 6A**). These RNA-specific bonds should directly contribute to the higher affinity of the CSD^YB-1^ to RNA compared to DNA.

**Fig 7:**
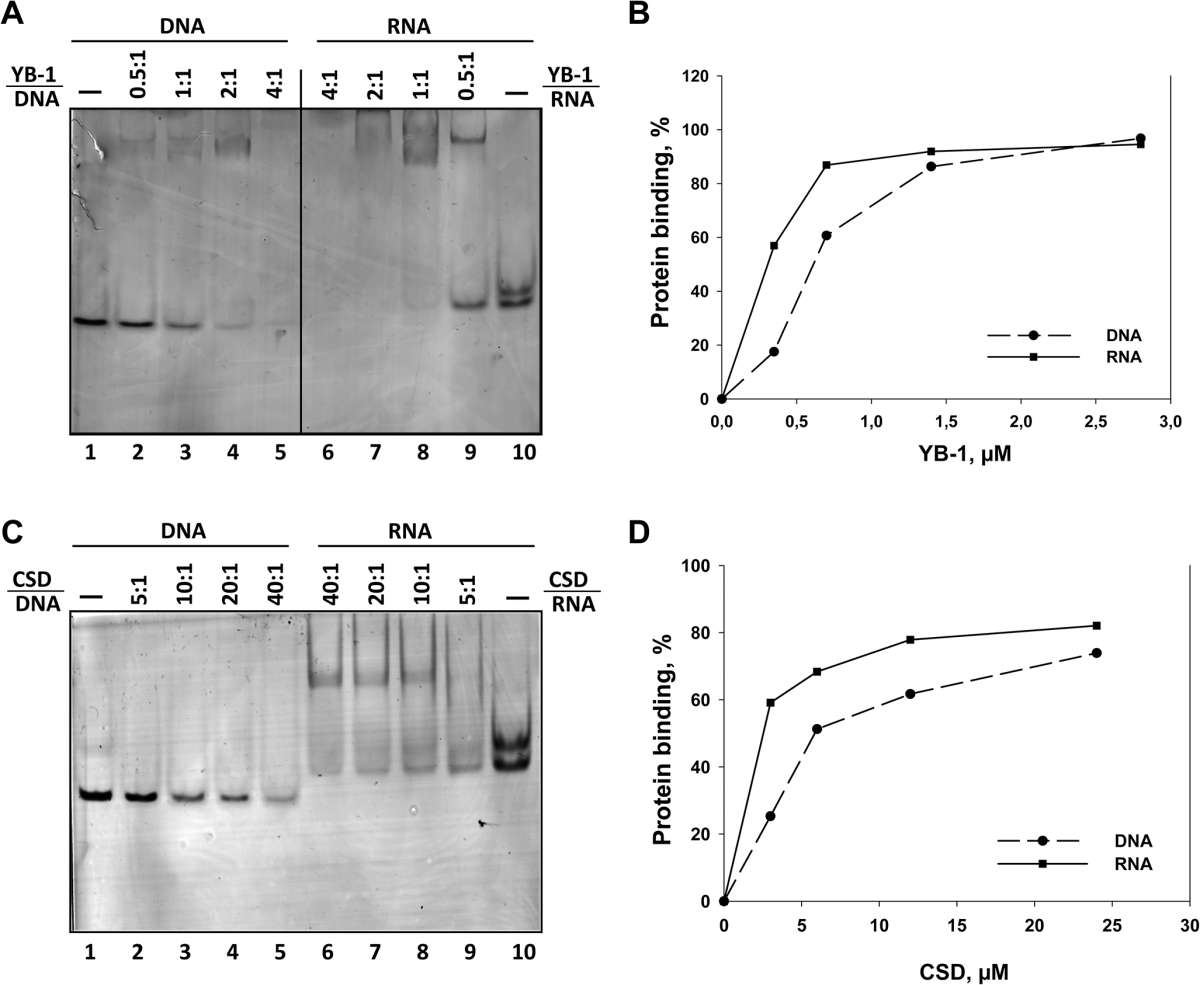
Determination of the apparent dissociation constants (Ka) of the complexes of recombinant YB-1 (A,B) and CSD (C,D) with RNA and DNA. A and C, Chemically synthesized RNA or DNA fragments of similar sequence and length (7 pmol) were incubated with increasing amounts of recombinant YB-1 (A) or CSD (C) in 10 μl of reaction buffer. The resultant RNA-protein or DNA-protein complexes were analyzed by 8% PAGE followed by silver staining. Protein/NA molar ratio are mentioned at the top of the figures for each experimental condition. B and D, Binding curves were drawn according to quantification of band intensities for free RNA or DNA using ImageJ software. The apparent dissociation constant (Ka) was estimated as protein concentration sufficient for 50% saturation. Solid and dotted lines show the RNA-protein and DNA-protein complex, respectively.

To verify this hypothesis, we evaluated the affinity of CSD^YB-1^ to G-rich DNA (5’-AGGATGGGTGAGTGAGGTAG-3’) and RNA (5’-AGGAUGGGUGAGUGAGGUAG-3’) oligonucleotides *in vitro*. Both oligonucleotides were incubated with increasing amount of recombinant full-length YB-1 or CSD^YB-1^. Quantification of the band intensity of each complex allowed estimating the apparent dissociation constants (**Fig 7 and S7 Fig**). Both YB-1 (**Fig 7A and 7B**) and CSD^YB-1^ (**Fig 7C and 7D**) bind RNA with more affinity than DNA: K_D_ was 0.3 μM and 0.6 μM for the YB-1:RNA and the YB-1:DNA complexes, respectively. K_D_ of CSD^YB-1^ complexes with RNA and DNA were one order of magnitude lower: 2.5 μM and 6 μM, respectively. These results are in line with our predictions from MDS.

Altogether, these data show that the CSD^YB-1^ exhibits a preferential binding to G-rich DNA and RNA sequences. This observation is also in agreement with other experimental data that showed that full-length YB-1 possesses a higher affinity for polyG over polyU and polyA [14]. These data strongly suggest that similarly to what proposed for ssDNA, the CSD^YB-1^ is the main contributor that defines the YB-1 binding specificity to RNA. The significant higher affinity of the CSD^YB-1^ for RNA can be explained by the presence of numerous H-bonds between the ribose and the CSD^YB-1^. Finally, the presence of intra-molecular RNA H-bonds may favor the adoption of a local conformation that facilitates either the binding of RNA to the CSD^YB-1^ or the stability of the complex. It is worth mentioning that the dramatic change in stability observed between CSD^YB-1^:ssDNA and CSD^YB-1^:ssRNA was not experimentally found in bacterial CSP [28]. Contrary to the CSD^YB-1^:sRNA complexes where numerous H-bonds within the RNA oligonucleotide stabilized the interaction with the CSD^YB-1^, such intramolecular H-bonds were not observed in the RNA oligonucleotides in complex with bacterial CSP. Therefore, these results re-emphasized a potential role of intramolecular H-bonds in the RNA oligonucleotide as a mechanism that favor the stability of CSD^YB-1^:ssRNA complexes.

### The CSD^YB-1^ binds to nucleic acid in a strand orientation-specific fashion

Structural analysis suggests that there is no physical limit for the CSD^YB-1^ to bind nucleic acids in either direct or reverse orientations (the “direct” orientation refers here as it appears in the bacterial CSP:oligo(dT) complexes 2ES2 and 2HAX). However it has been shown that all known OB (oligonucleotide/oligosaccharide binding)-fold proteins bind NA in a specific orientation, either direct or reversed [23]. To address the strand orientation specificity of the CSD^YB-1^ binding to nucleic acids, the most stable CSD^YB-1^:oligonucleotide complexes in both possible orientations were analyzed by MDS (**Fig 6B and 6C**). These oligonucleotides contained at positions 2 to 4, a GGT triplet for the DNA oligonucleotides, and a GGU or a UUU triplet for the RNA oligonucleotides. The MDS revealed that most of the inter-molecular contacts (stacking interactions and H-bonds) in the complexes containing an oligonucleotide in reverse orientation were weaker when compared to the same complexes in which the oligonucleotides were in direct orientation (**Table 1, S2, S5 and S6 Figs**). Accordingly, i) the distance between corresponding nucleotide bases and amino acids residues was significantly higher (P < 0.001), ii) the number of intermolecular H-bonds was lower except for CSDYB-1:oligo(Ur), iii) fluctuations of protein residues and oligonucleotide bases were significantly higher (P < 0.001), iiii) RMSD of protein and nucleotides backbone atoms were higher, when the oligonucleotide was reversed. The increased number of H-bonds for reversed U9 oligonucleotide was due to nonspecific interactions since the initial intermolecular contacts were dissociated very rapidly at the beginning of the trajectories, as illustrated by a distance > 12 Å median distance between the corresponding residues at the stacking sites over the entire length of the MDS (**Table 1**).

**Fig 8:**
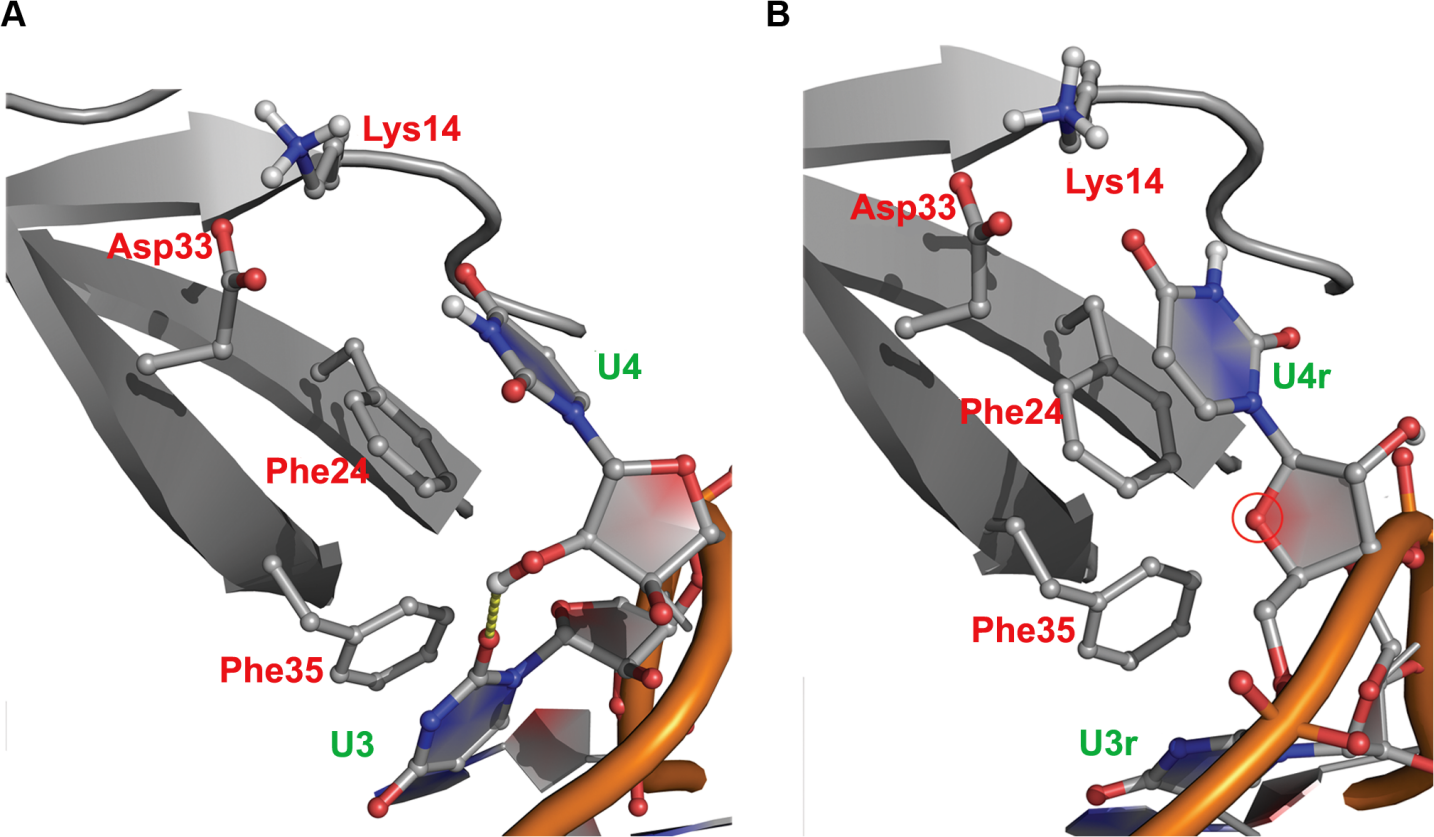
Interaction with the CSD^YB-1^ and the position 4 of direct- (A) or reverse-oriented (B) RNA oligonucleotides (orange). **A.** In the direct orientation, the *O2'* atom of U4 forms an H-bond with the *O2* atom of U3 (yellow dotted line). **B.** In contrast, the *O4'* atom of U4r does not form any H-bond, neither with other RNA atom nor with water molecule.

Analysis of MDS trajectories showed that reversing the oligonucleotide orientation entails unfavorable local interactions between the oligonucleotides tested and the CSD^YB-1^. Although these unfavorable interactions were observed at every position on the oligonucleotide, the most striking effect was observed for the binding site N4. For instance, when the CSD^YB-1^ binds to an oligo(U) in direct orientation, the *O2'* atom of U4 establishes a stable H-bond with the *O2* atom of U3 (**Fig 8A**). In contrast, when the binds an oligo(U) in reverse orientation (rU), the *O4'* atom of rU4 cannot establish any H-bond neither with atoms from neighboring nucleotides nor with water molecules (**Fig 8B**). Consequently, the system compensates the loss of H-bonds between the rU4r and the N4 site by interacting with water molecules. Under these conditions, the binding of oligo(U) to the CSD^YB-1^ is outcompeted by water molecules, which results in the dissociation of the complex and observed increasing the distance between binding sites and respective nucleotide bases. The strand specificity of the CSD^YB-1^:NA interaction was more prominent when the CSD^YB-1^ was bound to reverse oriented G-rich DNA compared with G-rich RNA. In this case, the stacking interactions were weakening more dramatically: the median distance for G-nucleotides at sites 2 was increased from 4.9Å to 7.1Å in the case of DNA and from 4.8Å to 5.2Å in the case of RNA; for the binding site 3 we observed increasing from 4.2Å to 5.2Å for DNA and similar medians of 4.3Å for RNA (**Table 1**). All increments were statistically significant.

If the estimations of free energies of binding between the different DNA and RNA oligos are in good agreement with experimental data as we were able to demonstrate, difference between direct and reverse orientations of DNA and RNA oligos (especially G-rich ones) are more difficult to interpret due to the limitations of the solvation of Poisson-Boltzmann equations by the MM-PBSA method (**S2 Table**). This method requires a representative sampling of the conformational space of the solute. In the case of highly flexible molecules like the reverse oriented oligos (see RMSD plots in **S4**and **S5 Figs**), the rapid dissociation from the CSD protein after few picoseconds of MDS, limits the portion of conformational space to sample. In addition, empirical molecular mechanics (MM) force fields like we used in MM-PBSA calculations, cannot described like quantum chemistry methods, higher order components of non-bonded attractive interactions involved in aromatic molecules stacking. If geometrical parameters of such stacking interactions are well rendered, MM methods used in MM-PBSA calculations penalize the most stable oligos like G-rich ones by underestimating their stability. However, combined with the other geometrical and physical parameters we used in this study, and keeping in mind the above-mentioned limitations, MM-PBSA method remains a good tool for ranking oligo/protein affinities.

Altogether, these data indicate at least one order of magnitude higher affinity of CSD^YB-1^ for direct orientated oligonucleotides which is in good agreement with the previously published experimental data for bacterial CSPs [26,27].

## Conclusion

MDS of different complexes between the CSD^YB-1^ and different 9-mer oligonucleotides allowed us to document the sequence specificity of the CSD^YB-1^ binding to nucleic acids and provided molecular insight into the differential affinity of the CSD^YB-1^ for RNA and DNA, in a sequence and strand orientation-specific manner. The differences between the NA binding sites of the CSD^YB-1^ and the bacterial CSPs contribute to understand the G-rich binding preference of YB-1 that was previously observed [14,25]. Furthermore, these data support a model whereby the binding specificity of YB-1 can be mainly attributed to its CSD rather than other domains of the protein. Interestingly, the CSD^YB-1^ exhibits a significantly lower affinity for nucleic acids than bacterial CSPs. However, contrary to the bacterial proteins that are solely composed of a CSD, YB-1 also contains two additional domains that also contribute to its interaction with nucleic acids [46] and possibly compensate for the relatively low affinity of the CSD for nucleic acids. Unfortunately, the absence of available structure for the other domains of YB-1 prevents the full-length protein to be studied by MDS. MDS also unraveled the role of the 2'-OH of the nucleic acid sugar moiety in stabilizing the interaction between the CSD^YB-1^ and RNA molecules. The presence of the 2'-OH in ribose contributed in the establishment of CSD^YB-1^:RNA H-bounds along with the formation of intra-molecular H-bounds that could maintain RNA molecules in a more favorable conformation than DNA upon binding to the CSD^YB-1^. In conclusion, MDS of the CSD^YB-1^ in interaction with nucleic acids appears as a valuable model to predict the binding properties of YB-1 for nucleic acids and understand its complex involvement in transcription and translation.

## Supporting Information

S1 Fig Correlation plots between experimental and calculated NMR chemical shifts obtained from different structures for ^13^C_α_ (left) and ^13^C_β_ (right) atoms of the human CSD^YB-1^: initial structure available in PDB (top), the last point structure after simulation (bottom). (TIF)

S2 Fig Ramachandran plots of human the CSD^YB-1^ allow to check the stereochemical quality of the models at different stages along MD trajectories: initial structure (left) and the structure after 50 ns simulation (right). The percentage of the amino-acid residues localized in the most preferable regions of the diagram is shown for each model.
(TIF)

S3 Fig Atomic fluctuations (RMSF) of the protein Cα-atoms along the MD trajectories starting from the beginning of the 50 ns simulation. The highest fluctuations are observed for the less stable complexes due to disruption of the intermolecular contacts.
(TIF)

S4 Fig RMSF mapped on the 3D structures of all the CSD^YB-1^:oligo complexes (DNA and RNA respectively at the top and the bottom of the figure). In red, highly flexible regions and in blue, the less flexible ones.
(TIF)

S5 Fig DNA nucleotides. Root mean square deviation (RMSD) for all atoms of CSD^YB-1^:oligonucleotide along the MD trajectories starting from the beginning of the 50 ns simulation. Plots are average values of three independent run of MDS. The highest fluctuations are observed for the less stable complexes.
(TIF)

S6 Fig RNA nucleotides. Root mean square deviation (RMSD) for all atoms of CSD^YB-1^:oligonucleotide along the MD trajectories starting from the beginning of the 50 ns simulation. Plots are average values of three independent run of MDS. The highest fluctuations are observed for the less stable complexes.
(TIF)

S7 Fig Electrophoretic analysis of YB-1 and CSD used in EMSA. Before and after 15 minute incubation at 30°C under EMSA conditions and subjected to 13% SDS-PAGE in a tris-tricine buffer system and stained with Coomassie brilliant blue of 1 μg of YB-1 (lane 1 and 2 respectively) and 0.5 μg of CSD (lane 3 and 4 respectively).
(TIF)

S1 Table H-bonds network between different nucleotides and amino acids at the YB-1 CSD surface. (DOCX)

S2 Table MM/PBSA calculations results (the solute dielectric constant is equal to 4). (DOCX)

S3 Table Interactions with participation of OH groups of ribose. (DOCX)
