# Supplementary figures and images for: The Cold Shock Domain of YB-1 Segregates RNA from DNA by Non-Bonded Interactions
Source: PLoS One. 2015 Jul 6;10(7):e0130318. doi: 10.1371/journal.pone.0130318 (PMC4493011; doi:10.1371/journal.pone.0130318)

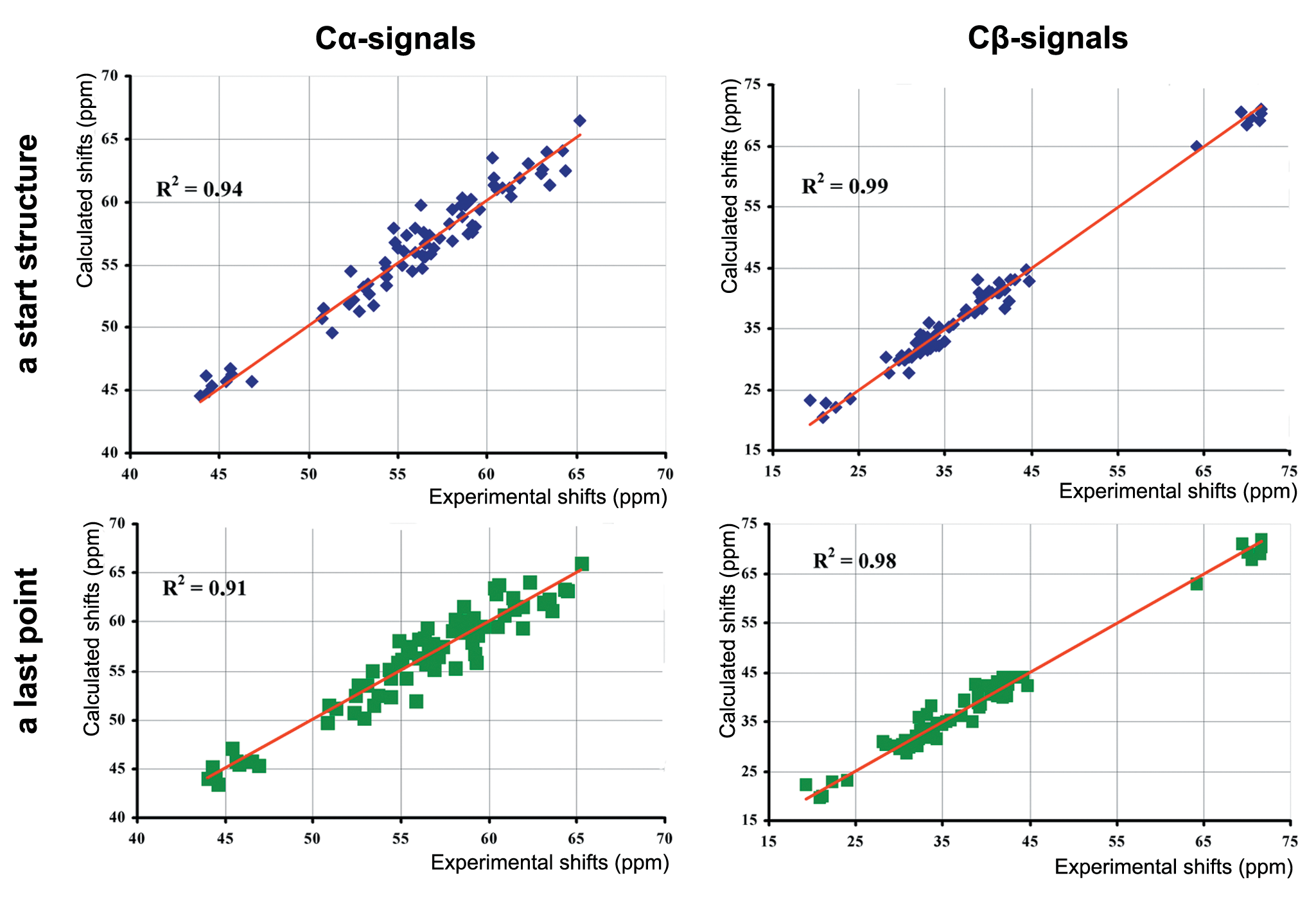

Supplement: S1 Fig — (TIF) [file pone.0130318.s001.tif]

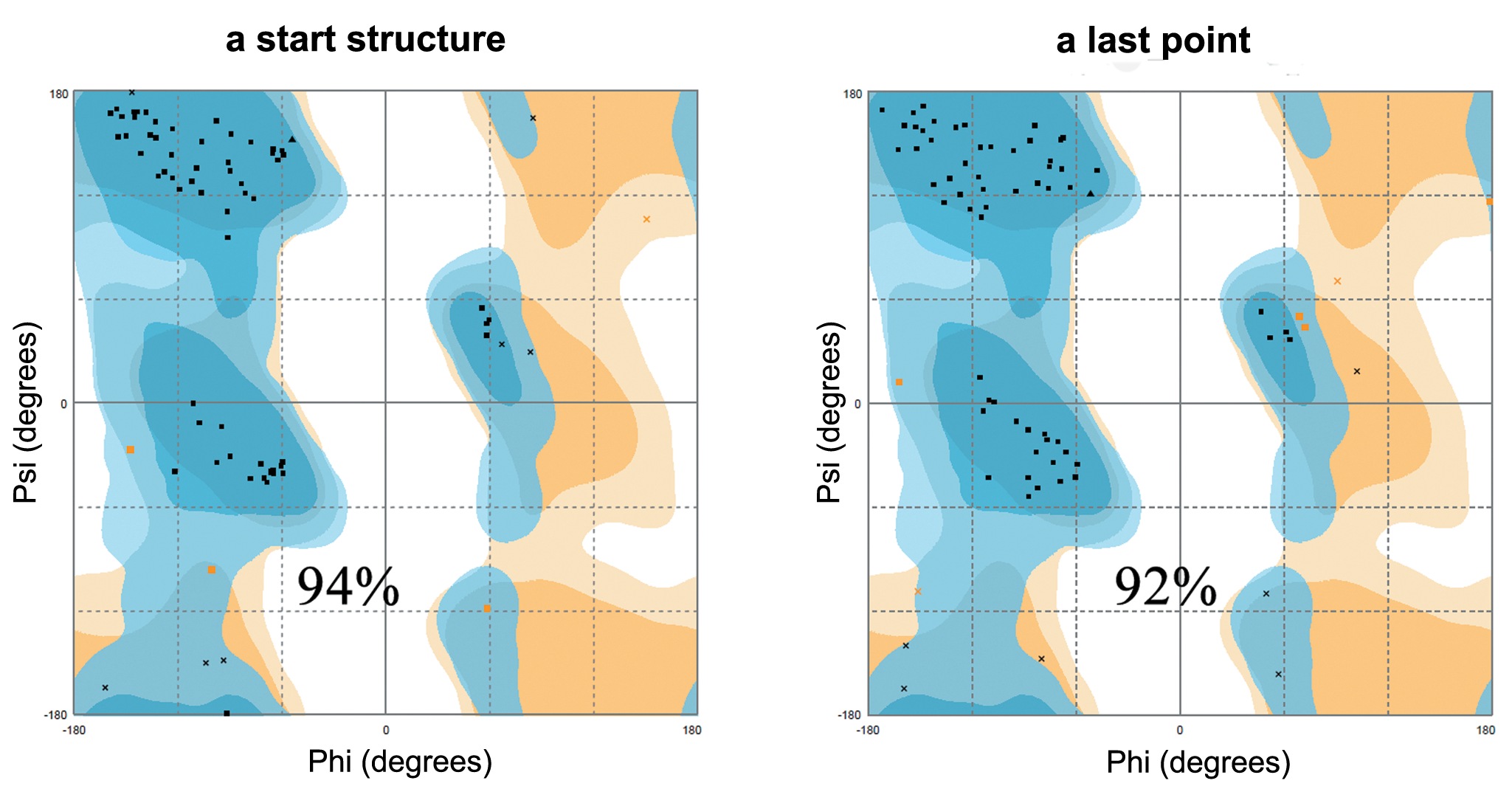

Supplement: S2 Fig — The percentage of the amino-acid residues localized in the most preferable regions of the diagram is shown for each model. (TIF) [file pone.0130318.s002.tif]

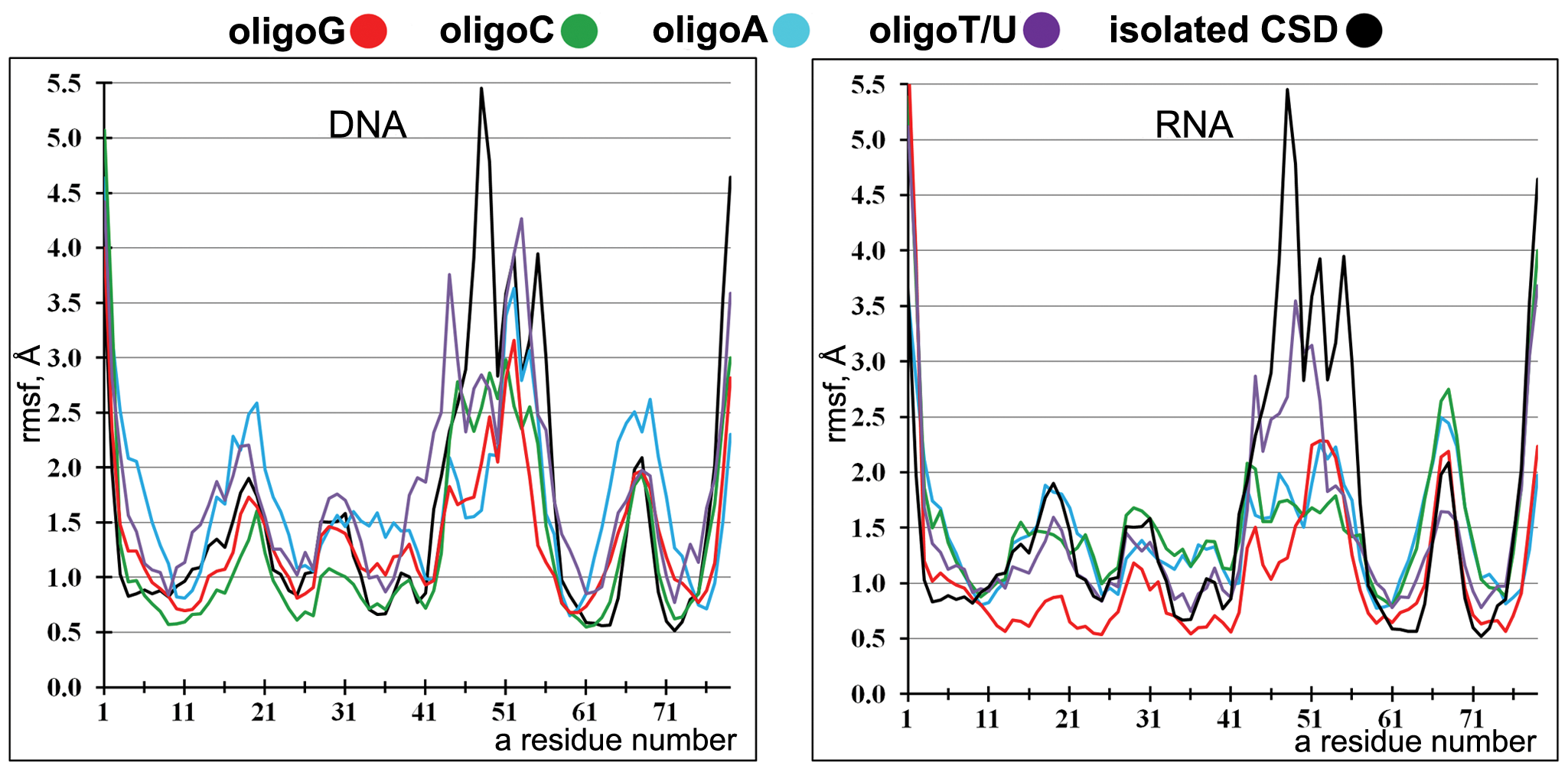

Supplement: S3 Fig — The highest fluctuations are observed for the less stable complexes due to disruption of the intermolecular contacts. (TIF) [file pone.0130318.s003.tif]

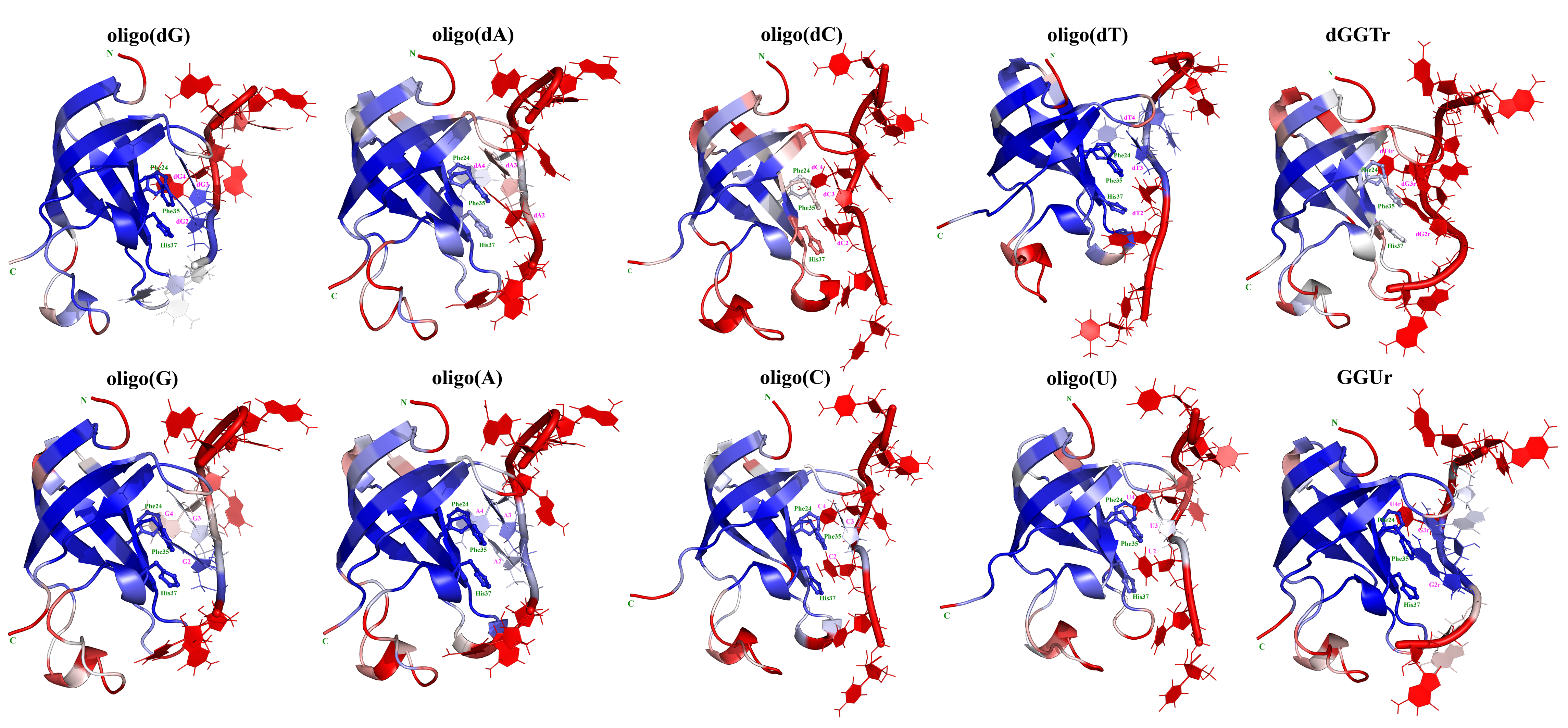

Supplement: S4 Fig — In red, highly flexible regions and in blue, the less flexible ones. (TIF) [file pone.0130318.s004.tif]

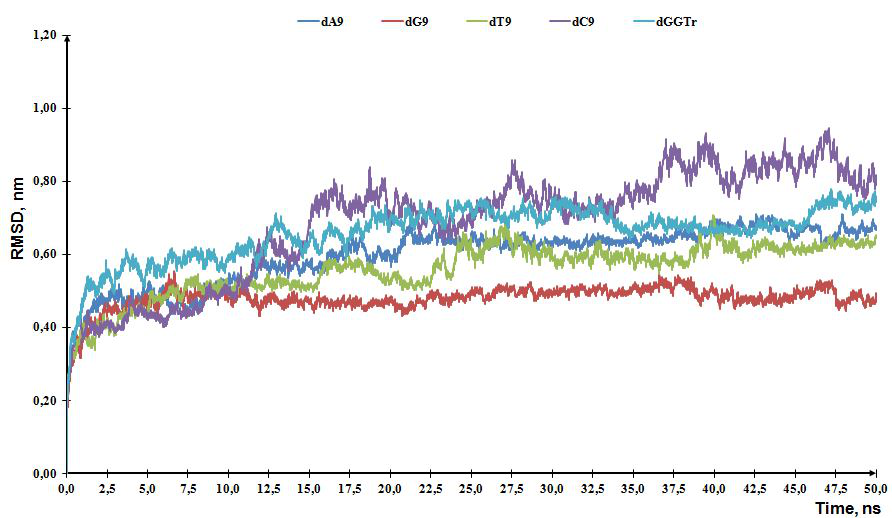

Supplement: S5 Fig — Root mean square deviation (RMSD) for all atoms of CSDYB-1:oligonucleotide along the MD trajectories starting from the beginning of the 50 ns simulation. Plots are average values of three independent run of MDS. The highest fluctuations are observed for the less stable complexes. (TIF) [file pone.0130318.s005.tif]

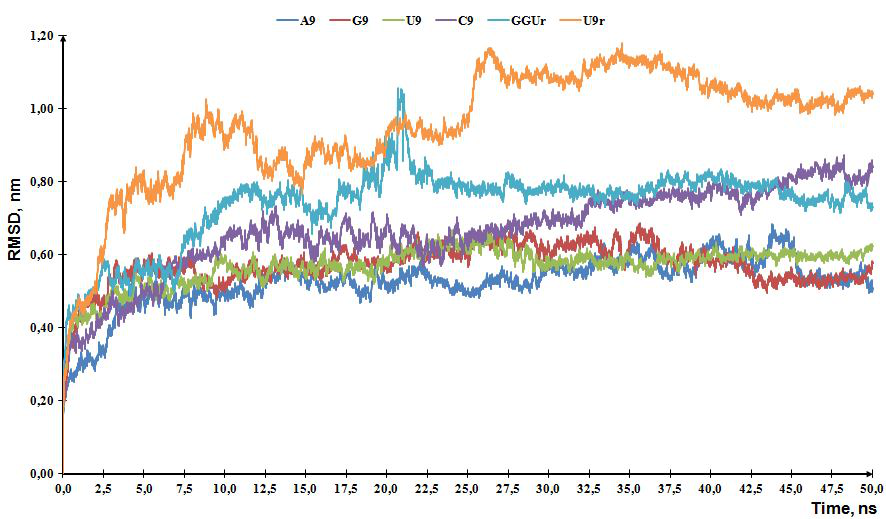

Supplement: S6 Fig — Root mean square deviation (RMSD) for all atoms of CSDYB-1:oligonucleotide along the MD trajectories starting from the beginning of the 50 ns simulation. Plots are average values of three independent run of MDS. The highest fluctuations are observed for the less stable complexes. (TIF) [file pone.0130318.s006.tif]

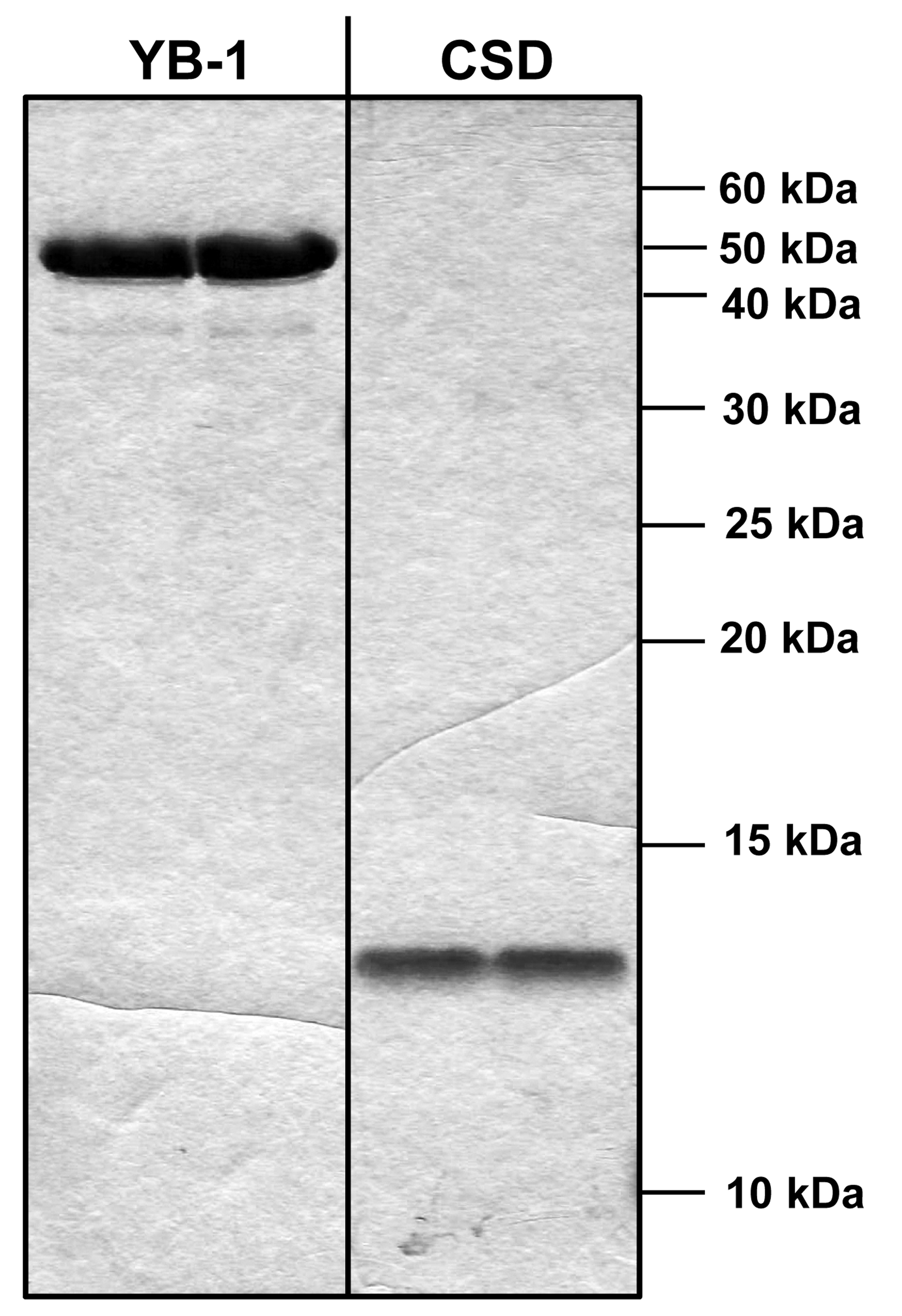

Supplement: S7 Fig — Before and after 15 minute incubation at 30°C under EMSA conditions and subjected to 13% SDS-PAGE in a tris-tricine buffer system and stained with Coomassie brilliant blue of 1 μg of YB-1 (lane 1 and 2 respectively) and 0.5 μg of CSD (lane 3 and 4 respectively). (TIF) [file pone.0130318.s007.tif]
