# Supplementary material for: The Cold Shock Domain of YB-1 Segregates RNA from DNA by Non-Bonded Interactions
Source: PLoS One. 2015 Jul 6;10(7):e0130318. doi: 10.1371/journal.pone.0130318 (PMC4493011; doi:10.1371/journal.pone.0130318)
Supplement: S1 Table — (DOCX) [file pone.0130318.s008.docx]

**Supporting Information**

**PONE-D-14-54873**

**The Cold Shock Domain of YB-1 segregates RNA from DNA by non-bonded interactions**

Vladislav G. Kljashtorny, Stanislav V. Nikonov, Lev P. Ovchinnikov, Dmitry N. Lyabin, Nicolas Vodovar, Patrick A. Curmi, Philippe Manivet

**Table S1**. H-bonds network between different nucleotides and amino acids at the YB-1 CSD surface.

| **Nucleotide binding site 2** | | | | | **Nucleotide binding site 3** | | | | | **Nucleotide binding site 4** | | | | |
| --- | --- | --- | --- | --- | --- | --- | --- | --- | --- | --- | --- | --- | --- | --- |
| Nucleotide atom* | Protein atom | SA, Å^2^ | Occ, % | Relative strength | Nucleotide atom | Protein atom | SA, Å^2^ | Occ, % | Relative strength | Nucleotide atom | Protein atom | SA, Å^2^ | Occ, % | Relative strength |
| A2 O4' | His37 ND1-H | 0.9 | 10 | 0.10 | A3 O5'  A3 O2P  A3 O3' | Lys68 NZ-H  Lys68 NZ-H  Ty22 OH-H | 8.2  24.2  21.2 | 15  45  11 | 0.13  0.23  0.06 | A4 O2P | Ty22 OH-H | 31.5 | 79 | 0.29 |
| C2 O1P | Asn20 ND2-H | 23.8 | 27 | 0.14 | C3 O2P  C3 N4-H | Lys68 NZ-H  Ala70 O | 24.2  21.1 | 25  12 | 0.13  0.07 | C4 n/d** | n/d | n/d | n/d | n/d |
| G2 N2-H  G2 N1-H  G2 O4' | Glu71 OE1/OE2  Glu71 OE1/OE2  His37 ND1-H | 16.0/15.8  19.7/19.5  0.0 | 73/79  54/61  32 | 0.50/0.54  0.33/0.37  0.32 | G3 N1-H | Lys68 O | 11.1 | 14 | 0.11 | G4 O2P | Ty22 OH-H | 32.0 | 58 | 0.21 |
| U2 O4'  U2 O2 | Asn17 ND2-H  Ty22 OH-H | 3.7  12.5 | 22  64 | 0.20  0.48 | U3 O2 | Lys68 NZ-H | 17.8 | 40 | 0.26 | U4 N3-H  U4 O4 | Asp33 OD1/OD2  Lys14 NZ-H | 8.6/12.4  8.4 | 37/60  75 | 0.31/0.45  0.62 |
| dA2 O4' | His37 ND1-H | 1.3 | 36 | 0.35 | dA3 N6-H  dA3 O2P | Ala70 O  Lys68 NZ-H | 17.3  33.1 | 29  22 | 0.19  0.07 | dA4 N6-H | Tp15 O | 18.5 | 21 | 0.13 |
| dC2 O4' | His37 ND1-H | 0.0 | 23 | 0.23 | dC3 O2P  dC3 O3' | Lys68 NZ-H  Lys68 NZ-H | 17.7  5.2 | 54  31 | 0.35  0.28 | dC4 N4-H | Ty22 OH | 15.9 | 89 | 0.61 |
| dG2 N1-H  dG2 N2-H  dG2 O4' | Glu71 OE1/OE2  Glu71 OE1/OE2  His37 ND1-H | 16.4/14.0  21.0/18.6  0.1 | 75/78  44/63  46 | 0.50/0.56  0.26/0.40  0.46 | dG3 O4' | Lys68 NZ-H | 14.3 | 16 | 0.11 | dG4 O1P  dG4 O1P | Ty22 OH-H  Asn20 ND2-H | 34.5  21.4 | 34  32 | 0.11  0.18 |
| dT2 O3'  dT2 O4'  dT2 N3-H  dT2 N3-H | Lys68 NZ-H  His37 ND2-H'  Glu71 OE1/OE2  Glu67 OE1/OE2 | 4.3  3.2  21.6/21.1  26.4/26.5 | 19  10  11/13  12/12 | 0.17  0.09  0.06/0.08  0.06/0.06 | dT3 O4'  dT3 O5' | Ty22 OH-H  Lys68 NZ-H | 4.2  5.5 | 49  20 | 0.45  0.18 | dT4 O2P  dT4 O1P  dT4 O3' | Lys68 NZ-H  Ty22 OH-H  Ty22 OH-H | 21.2  37.2  16.8 | 36  60  18 | 0.21  0.15  0.12 |

* A, C, G, T: ribonucleotides; dA, dC, dG, dT: deoxyribonucleotides.

** n/d - no H-bond with occupancy more than 10% were found.
