# Supplementary material for: The Cold Shock Domain of YB-1 Segregates RNA from DNA by Non-Bonded Interactions
Source: PLoS One. 2015 Jul 6;10(7):e0130318. doi: 10.1371/journal.pone.0130318 (PMC4493011; doi:10.1371/journal.pone.0130318)
Supplement: S2 Table — (DOCX) [file pone.0130318.s009.docx]

**Supporting Information**

**PONE-D-14-54873**

**The Cold Shock Domain of YB-1 segregates RNA from DNA by non-bonded interactions**

Vladislav G. Kljashtorny, Stanislav V. Nikonov, Lev P. Ovchinnikov, Dmitry N. Lyabin, Nicolas Vodovar, Patrick A. Curmi, Philippe Manivet

**S2 Table**. MM/PBSA calculations results (the solute dielectric constant is equal to 4).

| Oligo* | ΔE_elec_, kcal/mol | ΔE_vdw_, kcal/mol | ΔE_gas_ =  E_elec_ + ΔE_vdw_, kcal/mol | ΔG_PB_, kcal/mol | ΔG_SA,_ kcal/mol | ΔG_sol_ =  ΔG_PB_+ ΔG_SA,_ kcal/mol | ΔE_gas_ + ΔG_sol,_ kcal/mol | TΔS, kcal/mol | ΔG_bind_^,^ kcal/mol |
| --- | --- | --- | --- | --- | --- | --- | --- | --- | --- |
| **The complexes of CSD with DNA** | | | | | | | | | |
| dG9 | -324 ± 73 | -65 ± 9 | -389 ± 78 | 550 ± 60 | -9 ± 1 | 541 | 152 | -44 | 196 |
| dGGTr** | -228 ± 47 | -47 ± 9 | -274 ± 50 | 441 ± 31 | -8 ± 1 | 433 | 159 | -44 | 203 |
| dA9 | -196 ± 58 | -70 ± 12 | -266 ± 56 | 431 ± 56 | -10 ± 1 | 421 | 155 | -49 | 204 |
| dT9 | -195 ± 64 | -63 ± 11 | -258 ± 65 | 442 ± 46 | -10 ± 1 | 432 | 174 | -49 | 223 |
| dGGT | -215 ± 55 | -54 ± 9 | -269 ± 53 | 456 ± 54 | -8 ± 1 | 448 | 179 | -47 | 228 |
| dC9 | -234 ± 97 | -56 ± 11 | -290 ± 105 | 493 ± 83 | -8 ± 1 | 485 | 195 | -45 | 240 |
| dG9r | -258 ± 61 | -45 ± 7 | -303 ± 61 | 354 ± 36 | -8 ± 1 | 348 | 45 | -50 | 95 |
| **The complexes of CSD with RNA** | | | | | | | | | |
| G9 | -208 ± 65 | -92 ± 9 | -300 ± 66 | 396 ± 34 | -12 ± 1 | 384 | 84 | -41 | 125 |
| GGUr | -243 ± 56 | -59 ± 12 | -302 ± 59 | 416 ± 30 | -10 ± 1 | 406 | 104 | -47 | 151 |
| UCU | -151 ± 51 | -46 ± 9 | -198 ± 50 | 352 ± 40 | -7 ± 1 | 345 | 147 | -47 | 194 |
| A9 | -246 ± 86 | -71 ± 11 | -317 ± 88 | 489 ± 54 | -10 ± 1 | 479 | 162 | -53 | 215 |
| U9 | -204 ± 43 | -53 ± 9 | -258 ± 44 | 443 ± 40 | -8 ± 1 | 435 | 177 | -45 | 222 |
| GGU | -327 ± 119 | -61 ± 11 | -388 ± 125 | 569 ± 94 | -9 ± 1 | 560 | 172 | -50 | 222 |
| U9r | -300 ± 77 | -46 ± 9 | -345 ± 77 | 536 ± 86 | -7 ± 1 | 529 | 184 | -52 | 236 |
| C9 | -184 ± 58 | -45 ± 7 | -230 ± 61 | 481 ± 78 | -7 ± 1 | 474 | 244 | -47 | 291 |
| G9r | -266 ± 48 | -77 ± 8 | -343 ± 50 | 478 ± 27 | -11 ± 1 | 467 | 124 | -40 | 164 |
| **The complex CSP/dT7** | | | | | | | | | |
| dT7 | -120 ± 40 | -70 ± 5 | -190 ± 39 | -365 ± 20 | -9 ± 1 | -374 | -564 | -26 | -538 |

* A, C, G, T: ribonucleotides; dA, dC, dG, dT: deoxyribonucleotides.

** The letter r after an oligo indicates reversed orientation.
