# Supplementary material for: The Cold Shock Domain of YB-1 Segregates RNA from DNA by Non-Bonded Interactions
Source: PLoS One. 2015 Jul 6;10(7):e0130318. doi: 10.1371/journal.pone.0130318 (PMC4493011; doi:10.1371/journal.pone.0130318)
Supplement: S3 Table — (DOCX) [file pone.0130318.s010.docx]

**Supporting Information**

**PONE-D-14-54873**

**The Cold Shock Domain of YB-1 segregates RNA from DNA by non-bonded interactions**

Vladislav G. Kljashtorny, Stanislav V. Nikonov, Lev P. Ovchinnikov, Dmitry N. Lyabin, Nicolas Vodovar, Patrick A. Curmi, Philippe Manivet

**Table S3**. Interactions with participation of OH groups of ribose.

| Olig | With CSD atoms | | | | With RNA atoms at positions 2 to 4 | | | |
| --- | --- | --- | --- | --- | --- | --- | --- | --- |
|  | RNA atom | Protein atom | Occ, % | Relative strength | RNA atom | RNA atom | Occ, % | Relative strength |
| G9 | G1 O2'-H  G5 O2'  G8 O2'-H  G8 O2'-H | Asn20 O  Lys68 NZ-H  Glu32 OE1/OE2  Glu65 OE1/OE2 | 88  13  12/8  6/4 | 0.86  0.07  0.05/0.02  0.02/0.02 | G2 O2'-H  G3 O2'-H  G3 O2'-H | G3 O2P  G5 N7  G4 O5' | 12  12  8 | <0.01  0.08  0.06 |
| U9 | U0 O2'  U0 O2'-H  U1 O2'  U2 O2'  U3 O2'  U4 O2'-H | Asp55 N-H  Asp55 OD1/OD2  Asn17 ND2-H  Tyr22 OH-H  Lys68 NZ-H  Lys68 O | 13  8/8  72  5  12  5 | 0.09  0.04/0.04  0.65  0.04  0.09  0.02 | U1 O2'-H  U1 O2'-H  U2 O2'-H  U2 O2'-H  U3 O2'-H  U3 O2'-H  U4 O2'-H  U4 O2'-H  U8 O2'-H | U2 O4'  U2 O5'  U3 O5'  U3 O4'  U4 O5'  U5 O4'  U5 O2P  U3 O2  U4 O2P | 59  85  95  42  81  8  14  8  41 | 0.57  0.78  0.88  0.38  0.75  0.06  0.05  0.05  0.18 |
| A9 | A1 O2'-H  A2 O2'  A6 O2'-H  A8 O2' | Asn20 O  Lys68 NZ-H  Phe16 O  Val18 N-H | 11  9  16  9 | 0.09  0.06  0.12  0.07 | A1 O2'-H  A1 O2'-H  A2 O2'-H  A3 O2'-H  A3 O2'-H  A3 O2'-H  A4 O2'-H  A4 O2'-H | A2 O5'  A2 O4'  A3 O2P  A4 O5'  A4 O4'  A5 O4'  A5 O5'  A5 O4' | 50  22  23  86  50  6  63  45 | 0.45  0.20  0.08  0.76  0.44  0.05  0.47  0.31 |
| C9 | C0 O2'-H  C0 O2'  C1 O2'  C1 O2'-H  C2 O2'  C5 O2'  C5 O2' | Glu38 OE1  Arg47 NH2-H  His37 ND1-H  Asn20 O  His37 ND1-H  Asn17 ND2-H  Asn20 ND2-H | 21  9  10  5  5  8  5 | 0.12  0.04  0.09  0.04  0.04  0.04  0.03 | C1 O2'-H  C1 O2'-H  C1 O2'-H  C2 O2'-H  C3 O2'-H  C3 O2'-H  C3 O2'-H  C4 O2'-H  C4 O2'-H  C4 O2'-H | C2 O4'  C2 O5'  C2 O1P  C3 O2P  C4 O5'  C4 O4'  C4 O2P  C5 O1P  C5 O4'  C5 O5' | 56  64  7  62  24  10  9  26  14  6 | 0.48  0.54  0.04  0.33  0.17  0.06  0.03  0.03  0.08  0.04 |
